# Glycolysis is reduced in dengue virus 2 infected liver cells

**DOI:** 10.1038/s41598-024-58834-w

**Published:** 2024-04-09

**Authors:** Chanida Chumchanchira, Suwipa Ramphan, Wannapa Sornjai, Sittiruk Roytrakul, Pathrapol Lithanatudom, Duncan R. Smith

**Affiliations:** 1 PhD Degree Program in Biology (International Program), Faculty of Science, Chiang Mai University, Chiang Mai, 50200 Thailand; 2 Institute of Molecular Biosciences, Mahidol University, Nakhon Pathom, 73170 Thailand; 3 National Center for Genetic Engineering and Biotechnology (BIOTEC), National Science and Technology Development Agency, Khlong Luang, Pathum Thani, 12120 Thailand; 4 Department of Biology, Faculty of Science, Chiang Mai University, Chiang Mai, 50200 Thailand

**Keywords:** Virology, Cellular microbiology, Mechanisms of disease

## Abstract

Infections with dengue virus (DENV) remain a worldwide public health problem. A number of bona fide cellular targets of DENV have been identified including liver cells. Despite the many lines of evidence confirming the involvement of hepatocytes during DENV infection, only a few studies have used proteomic analysis to understand the modulation of the cellular proteome occurring upon DENV infection. We utilized a 2D-gel electrophoresis analysis to identify proteins that were differentially regulated by DENV 2 infection of liver (Hep3B) cells at 12 h post infection (hpi) and at 48 hpi. The analysis identifies 4 proteins differentially expressed at 12 hpi, and 14 differentially regulated at 48 hpi. One candidate protein identified as downregulated at 48 hpi in the proteomic analysis (GAPDH) was validated in western blotting in Hep3B cells, and subsequently in induced pluripotent stem cell (iPSC) derived human hepatocytes. The reduced expression of GAPDH was coupled with an increase in NADH, and a significantly reduced NAD + /NADH ratio, strongly suggesting that glycolysis is down regulated in response to DENV 2 infection. Metformin, a well characterized drug used in the treatment of diabetes mellitus, is an inhibitor of hepatic gluconeogenesis was shown to reduce the level of DENV 2 infection and new virus production. Collectively these results show that although glycolysis is reduced, glucose is still required, possibly for use by the pentose phosphate pathway to generate nucleosides required for viral replication.

## Introduction

The last 80 years have seen an extensive and alarming spread of the mosquito transmitted dengue virus (DENV; family *Flaviviridae*, genus *Flavivirus*, species *Dengue virus*). Transmission of DENV to humans primarily occurs during the bite of an infected *Aedes* species female mosquito during a blood meal^1^. Currently DENV is endemic in around 100 tropical and semi-tropical counties around the world, causing an estimated 400 million new infections each year^2^. Of the 400 million infections, some 100 million of these show some degree of symptoms, while the remaining infections are largely asymptomatic^2^. Symptoms can range from classical dengue fever characterized by rash, headache, muscle and joint pain and pain behind the eyes, to the life-threatening dengue hemorrhagic fever (DHF) and dengue shock syndrome (DSS)^3^. A number of lines of evidence have suggested that the liver, including hepatocytes and kupffer cells is a major target organ of DENV infection. This is supported by increased levels of the liver enzymes alanine aminotransferase and aspartate aminotransferase^4^, increased rates of liver failure in severe DENV patients^5^, detection of infected hepatocytes in autopsy specimens from people who died from dengue infection^6^, and the infectability of primary human hepatocytes^7^.

DENV is a positive sense single-stranded enveloped RNA virus^8^. The viral genome encodes a polyprotein that encodes 3 structural proteins, namely, the capsid (C), pre-cursor membrane (prM) and envelope (E) proteins, and 7 non-structural proteins (NS1, NS2A, NS2B, NS3, NS4A, NS4B and NS5)^8^. The species *Dengue virus* contains four viruses, DENV 1, DENV 2, DENV 3 and DENV 4, that shared approximately 65% of their genome^9–11^. During infection the dengue virions bind to cell-surface receptors and are internalized through endocytosis in a clathrin-dependent manner^12^. The viral RNA is translated into a polyprotein prior to assembly at the endoplasmic reticulum and the immature virions are trafficked to the Golgi body where during passage they undergo furin-mediated cleavage of pre-membrane protein. Finally, the mature virions are released by exocytosis^13–15^. DENV has evolved several strategies to hijack and utilize host cell machinery to facilitate its replication cycle. Therefore, viruses modulate expression of host proteins to accomplish activities like providing energy for viral genome replication and manipulating cellular pathway to establish an optimal environment for replication^16^. In contrast, host cells induce protein expression to inhibit viral infections^17,18^. Eventually, the study of host protein changes during dengue infection may provide a beneficial understanding of viral production mechanism and reveal promising targets for the development of novel therapeutics. Only a few previous studies have investigated the protein changes during DENV infection of liver cells^19–21^, and these have indicated dysregulation of a number of processes including host metabolism and the apoptosis pathway.

Metformin (1,1-dimethylbiguanide hydrochloride) has been widely used as blood glucose-lowering by inhibiting gluconeogenesis in treatment of type 2 diabetes mellitus (T2DM) patients for 60 decades^22^. Notably, metformin has been shown to have pleiotropic activities against cancer^23^, inflammatory^24^, and also possesses a neuroprotective activity^25^. Interestingly an in vivo study showed that metformin potentially lowers the risk of severe dengue among diabetes patients that use metformin users when compared to non-users^26^. Therefore, metformin may be a promising anti-dengue agent. This study performed a proteomic study in a hepatocarcinoma cell line after infection with DENV 2 and validated the results in hepatocyte-like cells, and based on the results investigated the potential anti-DENV activity of metformin.

## Materials and methods

### Cells and viruses

Hep3B (human hepatocellular carcinoma) cells (ATCC HB-8064) were cultured in Dulbecco’s minimal essential medium (DMEM, Gibco, Invitrogen, Carlsbad, CA) supplemented with 10% heat-inactivated fetal bovine serum (FBS, Gibco, Invitrogen) and incubated at 37 °C with 5% CO_2_. Dengue virus serotype 2 (DENV 2; strain 16681) was propagated in *Aedes albopictus* C6/36 cells (ATCC CRL-1660) as previously described^27^. After propagation the virus supernatant was centrifuged at 1000xg to remove cell debris, and the supernatant was supplemented with 20% heat-inactivated fetal bovine serum and stock viruses were stored frozen at − 80 °C. Virus titer was determined by standard plaque assay on LLC-MK_2_ (Rhesus monkey kidney) cells (ATCC CCL-7) as described elsewhere^27^.

### Virus infection

Hep3B cells were seeded into six-well plates and cultured under standard growth condition for 24 h. After the cells reached ~ 70–80% confluence, the culture medium was removed, and the cells were mock infected or infected with DENV 2 at multiplicity of infection (MOI) of 1 for 2 h, after which the virus containing medium was removed and replaced by fresh culture medium containing FBS and cells were further incubated under standard condition for either 12 or 48 h.p.i. as appropriate. after which cells were pelleted by centrifugation. All experiments were undertaken independently as three biological replicates.

### Two-dimensional (2D)-gel electrophoresis

Cell pellets from mock- and DENV-infected Hep3B cells were lysed using RIPA buffer (1% NP-40, 0.5% sodium deoxycholate, 0.1% sodium dodecyl sulfate, 137 mM sodium chloride, 2.7 mM potassium chloride, 4.3 mM disodium hydrogen phosphate, 1.4 mM potassium dihydrogen phosphate) containing protein inhibitor cocktail (PIC) and proteins were precipitated overnight using acetone and methanol, after which the protein pellets were dissolved in lysis C buffer (8 M urea, 2 M thiourea, 4% CHAPS, 20 mM DTT, 1 mM PMSF, 1 mM benzamide) prior to determining the protein concentration by the Bradford assay. Subsequently, 250 µg of the purified proteins were loaded onto Immobiline Drystrips (pH 3–10 NL, 7 cm) containing 2% IPG buffer (Amersham Biosciences, Chalfont, United Kingdom) and 0.5% bromophenol blue and the strips were rehydrated for 12 h. Proteins were subjected to isoelectric focusing in a Multipor II electrophoresis system (Amersham Biosciences,) at the following voltages 300 V for 200 Vh, 1000 V for 300 Vh, a gradient to 3000 V for 4000 Vh, 5000 V for 4500 Vh and 5000 V for 3000 Vh. After focusing, the IPG strips were reduced in equilibration buffer (50 mM Tris–HCl (pH 8.8), 6 M urea, 30% v/v glycerol, 2% SDS w/v and 1% bromophenol blue) supplemented with 100 mM DTT for 15 min and then proteins were alkylated in equilibration buffer containing 150 mM iodoacetamide (IAA) for 30 min. The proteins were separated in the second dimension via 12.5% SDS-PAGE. Gels were stained with Coomassie Blue G250 after which the gels were visualized under a GS-900 calibrated Densitometer (Bio-Rad Laboratories, Hercules, CA). All experiments were undertaken as three independent biological replicates. Image data were analyzed using ImageMasterTM 2D Platinum version 7.0 software (Amersham Biosciences). Statistical analysis was performed by student’s t test with a p value of less than 0.05 being considered as statistically significant.

### Western blot assay

Purified protein samples from mock- and DENV-infected Hep3B cells were separated by 12% SDS-PAGE and subsequently transferred to nitrocellulose membranes. The protein containing membranes were probed with antibodies directed against glyceraldehyde-3-phosphate dehydrogenase (GAPDH), superoxide dismutase 1 (SOD1), stress induced phosphoprotein 1 (STIP1), DENV E protein (HB112), DENV NS1, DENV NS5 and actin followed by appropriate HRP-conjugated secondary antibodies with indicated dilution. Antibodies and dilutions are in Supplemental Table S1.

### NAD + /NADH assay

Hep3B cells were mock infected or infected with DENV 2 at MOI 1. The virus was removed before complete culture medium was added into cells, then the infected cells were further incubated under optimal condition for 48 h. The cells were harvested and lysed with NADH/NAD + extraction buffer (Abcam, Cambridge, United Kingdom). The sample lysates were added into 96-well plates and the NADH developer was added followed by measurement of the NAD + /NADH concentrations using a microplate absorbance reader at 450 nM.

### MTT assay

Hep3B cells were seeded into ninety-six well tissue culture plates and were cultured under standard conditions for 24 h. After the cells reached 80–89% confluence, the culture medium was removed, and cells were incubated at 37 °C with 5% CO_2_ with various concentration of metformin diluted in culture medium with an appropriate dilution of DMSO used as a control treatment. Cells viability was determined using the MTT (3-(4,5-dimethylthiozol-2-yl)-2,5-diphenyl tetrazolium bromide) assay (Merck, KGaA). At 24 h post-treatment, cells were incubated with MTT at 37 °C for 1 h followed by incubation with DMSO at 37 °C to solubilize the formazan product. The absorbance was measured using a microplate absorbance reader at 570 nM.

### Virucidal activity testing

Virus was incubated with various concentrations of metformin at 37 °C for 1 h after which the virus solution was tenfold serially diluted with BA-1 medium diluent (1X medium 199/Earle’s balanced salts, 0.05 M Tris–HCl pH 7.6), 1% serum albumin, 0.075% NaHCO_3_, and 100U of penicillin–streptomycin per mL). The virus titer after incubation was determined by plaque assay on LLC-MK_2_ cells as described elsewhere^27^.

### Flow cytometry

Hep3B cells were mock infected or infected with DENV 2 for 2 h, then the virus was removed. Then, cells were continuingly incubated with metformin in normal growth media at concentrations of 1, 5, and 10 mM and incubated under standard conditions. At 24 h post infection the cells were harvested and blocked with 10% normal goat serum on ice for 30 min. After that, the cells were fixed using 4% paraformaldyhyde and permeabilized by addition of 0.2% of Triton X-100 as described previously^19^. Subsequently, the cells were incubated with a pan specific mouse anti-dengue virus monoclonal antibody from hybridoma HB114 diluted 1:150 at 4 °C overnight. The cells were washed with 1% BSA in PBS-IFA (0.05 M NaH_2_PO_4_, 0.05 M Na_2_HPO_4_, 0.154 M NaCl) and incubated in a dark place with a FITC goat anti-mouse IgG antibody diluted 1:40 for 1 h. The fluorescence signal was detected by flow cytometry on a BD FACSCalibur cytometer (BD, Franklin Lakes, NJ) using CELLQuest software (version 3.3). All experiments were undertaken independently in triplicate.

### RNA extraction and quantitative reverse-transcription PCR

RNA was extracted from cell pellets from mock- and DENV-infected Hep3B cells at 12 and 48 h.p.i using Trizol reagent (Invitrogen, Thermo Fisher, Waltham, MA, USA) following the manufacturer’s protocol. 1 µg of total RNA was mixed with random hexamer primers (Invitrogen) and RevertAid Reverse Transcriptase for cDNA synthesis, followeing the manufacturer’s protocol. To detect the mRNA expression level, cDNA was used as a template to perform quantitative reverse-transcription PCR using KAPA SYBR FAST qPCR Master MIX (Merck, Darmstadt, Germany) with specific primers for GAPDH (GAPDH_F: 5ʹ–AGCCACATCGCTCAGACAC-3 and GAPDH_R: 5-GCCCAATACGACCAAATCC-3ʹ) and actin (Actin_F: 5ʹ–GAAGATGACCCAGATCATGT-3″ and Actin_R: 5ʹ–ATCTCTTGCTCGAAGTCCAG-3ʹ). The cycle conditions were 95 °C for 5 min, followed by 40 cycles of denaturing at 95 °C for 15 s, annealing at 55 °C for 20 s and extension at 72 °C for 20 s undertaken on a Eppendorf Realplex 4 qPCR Real time thermocycler. The relative expression was calculated by the normalization against the β-actin gene according to the following equation: ΔΔCt = ΔCt (sample)–ΔCt (mock). Experiments were performed independently in triplicate with triplicate qRT-PCR.

### Metformin treatment

Hep3B cells were seeded in six-well plates and cultured under standard growth conditions for 24 h. After the cells reached 60–70% confluence, the culture medium was removed, and the cells were mock infected or infected with DENV 2 for 2 h, after which the virus inoculum was removed and various concentration of metformin (Merck Group, Darmstadt, Germany) were added into the cells in normal growth medium and cells were then further incubated under standard conditions for 24 h. All experiments were undertaken as three independent biological three replicates.

### Culture of induced pluripotent stem cell (iPSC) derived human hepatocytes

The iPSC derived human hepatocytes (Definigen, Cambridge, England) were obtained as a cryopreserved sample and cells were recovered for 12 days following the manufacturer’s instructions. Briefly, cells were thawed on collagen-coated 24 well plates (Greiner Bio-One, Frickenhausen, Germany) at density of 5 × 10^5^ cells per well using minimum essential medium, supplemented with 2% non-essential amino acids, 2% chemical defined lipid concentrate (Thermo Fisher Scientific, Waltham, MA), 0.1% insulin (Sigma, Saint Louis, MO) and manufacturer’s cytokines. Cells were cultured in Hepatozyme medium (Invitrogen, Waltham, MA), supplemented with 2% non-essential amino acids, 2% chemical defined lipid concentrate (Thermo Fisher Scientific, Waltham, MA), 0.1% insulin (Sigma, Saint Louis, MO) and manufacturer’s cytokines, and were maintained at 37 °C with 20% O_2_.

### Immunofluorescence assay

Cells were fixed with 4% paraformaldehyde in 1X PBS then permeabilized with 0.3% Trition X-100 in 1X PBS. The cells were blocked with 5%BSA in 1X PBS and then probed with primary antibodies against albumin and dengue E protein. Subsequently, the cells were incubated with secondary antibodies conjugated with fluorescent labels and nuclei were stained with DAPI before detecting the signal under a ZEISS LSM 800 confocal microscope running ZEN (blue edition) version 2.3 (Carl Zeiss Microscope GmbH, Jena, Germany). Antibodies and dilutions are in Supplementary Table S2.

### Statistical analysis

All experiments were performed independently in triplicate with duplicate plaque assay. The results are displayed as mean ± SEM. The data were subsequently analyzed using the GraphPad Prism program (GraphPad Software Inc., San Diego, CA). Analysis was performed using the independent *t*-test of PASW Statistics 18.0.0 (SPSS Inc., Chicago, IL) and the statistical significance is shown with *p*-values of < 0.05*, < 0.01** and < 0.001***. EC_50_ and CC_50_ values were calculated using the freeware ED50plus (v1.0) software (http://sciencegateway.org/protocols/cellbio/drug/data/ed50v10.xls).

## Results

### Optimization of DENV 2 infection of Hep3B cells

We initially optimized the infection of Hep3B cells with DENV 2. To undertake this, Hep3B cells were infected with DENV at different MOIs, and at 48 and 72 h post-infection (h.p.i.) the level of infection was determined by flow cytometry. The results (Supplemental Fig. 1) showed that at 48 h.p.i cells were more than 80% infected at all MOIs investigated (1, 5, 10 and 20). By 72 h the level of infection had dropped slightly, possibly as a result of the induction of apoptosis in infected cells. We therefore selected MOI 1 as the optimal MOI, and reconfirmed this result (Supplemental Fig. 2). We additionally investigated the level of infection at MOI 1 at 12 h.p.i. and showed that there was a low, but clear degree of infection (Supplemental Fig. 3).

### 2D-gel analysis of differentiating host protein in Hep3B cells after DENV 2 infection

**Figure 1:**
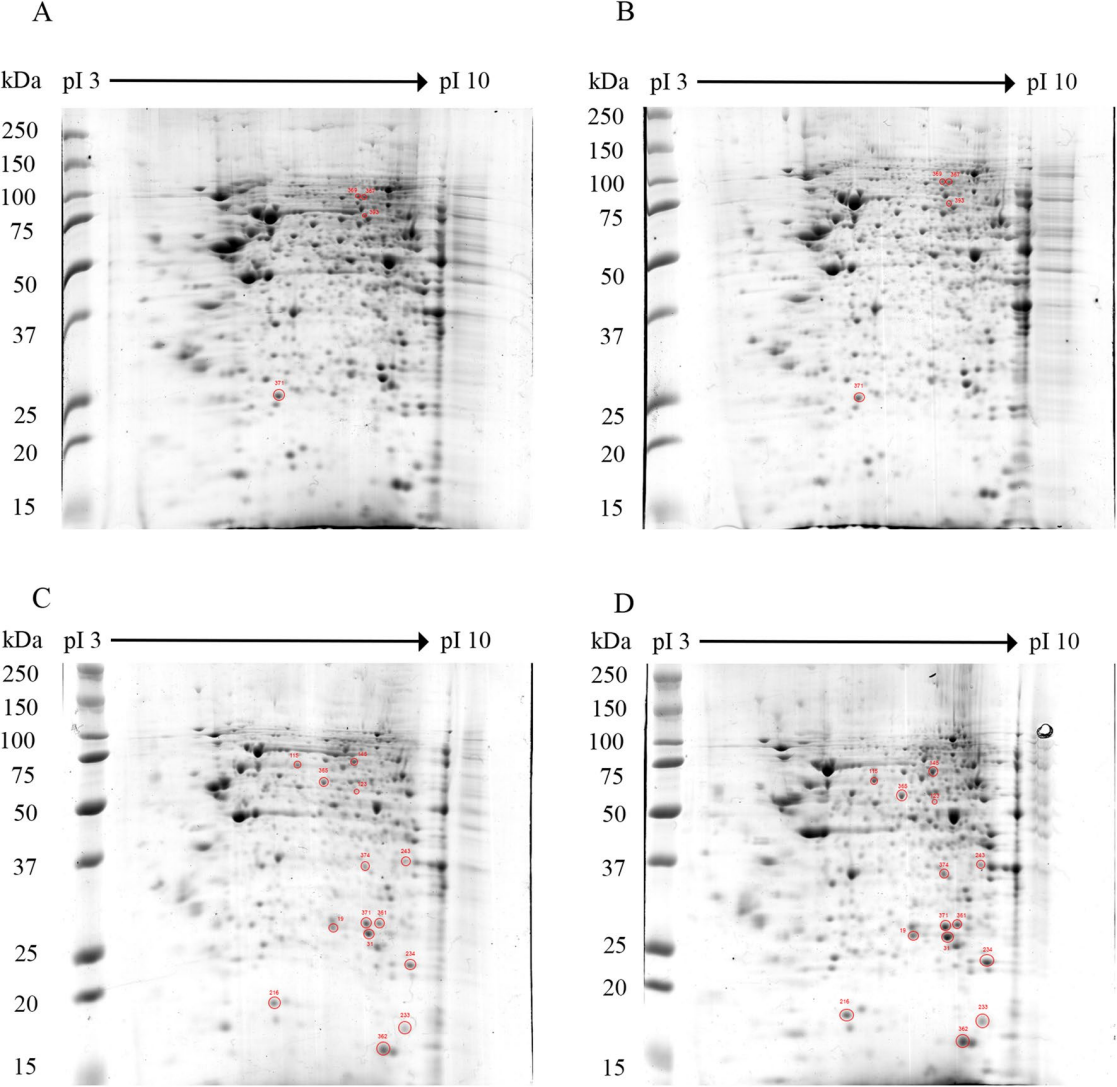
2-D proteomic analysis of mock infected or DENV 2 infected Hep3B cells. Representative two-dimensional polyacrylamide gels of Hep3B cells after 12 (**A**, **B**) or 48 h (**C**, **D**) of mock infection (**A**, **C**) or DENV 2 infection (**B**, **D**). All 2-D gels can be found in the supplemental materials (Supplemental Figs. S4-S7).

Hep3B cells were either mock infected or infected with DENV 2 for either 12 or 48 h. The experiment was undertaken as three independent biological replicates. The cell pellets were collected at the appropriate time and proteins were extracted. For proteomic analysis, protein samples were subjected to 2D-gel electrophoresis followed by staining with Coomassie Blue G250 (Fig. 1 and Supplemental Fig. S4-S7). The spots were analyzed and four differentially expressed spots were identified at 12 h post infection in infected samples as compared to mock infected, while 14 differentially expressed spots were detected at 48 h post infection in infection as compared to mock infection. The differentially expressed spots were excised from the gels and the proteins subjected to identification by in-gel tryptic digestion and subsequent analysis by mass spectrometry (Tables 1 and 2).

**Table 1:** List of proteins which were differentially expressed after Hep3B cells were infected with DENV 2 for 12 h as compared to mock infected cells.

Spot No	Protein name	pI	Uniprot accession	Score	Intensity			*P* value
					Mock infection	DENV 2 infection	Fold change	
393	Stress-induced-phosphoprotein 1	6.40	P31948	300	687.32	863.27	1.26	9.89E − 03
369	Threonine–tRNA ligase	6.23	P26639	352	1441.57	1438.75	1.00	1.22E − 02
367	Glutamine-fructose-6-phosphate aminotransferase [isomerizing] 1	6.66	Q06210	102.5	574.76	870.27	1.51	1.94E – 02
371	Glutathione S-transferase P	5.43	P09211	250.67	768.51	2779.05	3.62	2.64E − 02

**Table 2:** List of 14 proteins which were differentially expressed after Hep3B cells were infected with DENV 2 for 48 h as compared to mock infected cells.

Spot no	Protein name	pI	Uniprot accession	Score	Intensity			*P* value
					Mock infection	DENV 2 infection	Fold change	
371	Phosphoglycerate mutase 1	6.67	P18669	297	1129.40	690.05	0.61	3.79E − 04
365	T-complex protein 1 subunit beta	6.01	P78371	406	1953.53	491.37	0.25	3.61E − 03
361	Carbonic anhydrase 2	6.87	P00918	303	1238.21	1612.68	1.30	5.56E − 03
31	triosephosphate isomerase isoform 1	6.45	P60174	818	2072.52	2319.83	1.12	9.94E − 03
374	Aldo–keto reductase family 1 member B1	6.51	P15121	212	1539.12	2803.27	1.82	1.29E − 02
233	Cofilin-1	8.22	P23528	73	4501.44	2368.83	0.53	1.48E − 02
234	Peroxiredoxin-1	8.27	Q06830	488	8637.54	2912.59	0.34	1.91E − 02
216	Superoxide dismutase [Cu–Zn]	5.70	P00441	163	1315.74	528.01	0.40	2.01E − 02
362	Peptidyl-prolyl cis–trans isomerase A	7.68	P62937	283	2233.09	2090.24	0.94	2.03E − 02
243	Glyceraldehyde-3-phosphate dehydrogenase	8.57	P04406	122	4565.55	1917.50	0.42	2.19E − 02
19	Hypoxanthine–guanine phosphoribosyltransferase	6.21	P00492	187	3338.28	1415.07	0.42	2.61E − 02
115	T-complex protein 1 subunit alpha	5.80	P17987	504	3772.48	1328.12	0.35	3.14E − 02
123	Septin-11	6.36	Q9NVA2	137	4287.90	3170.40	0.74	3.17E − 02
145	Stress-induced-phosphoprotein 1	6.40	P31948	273	3116.70	2220.35	0.71	4.73E − 02

### Ontological analysis of differentially expressed host proteins after DENV 2 infection

**Figure 2:**
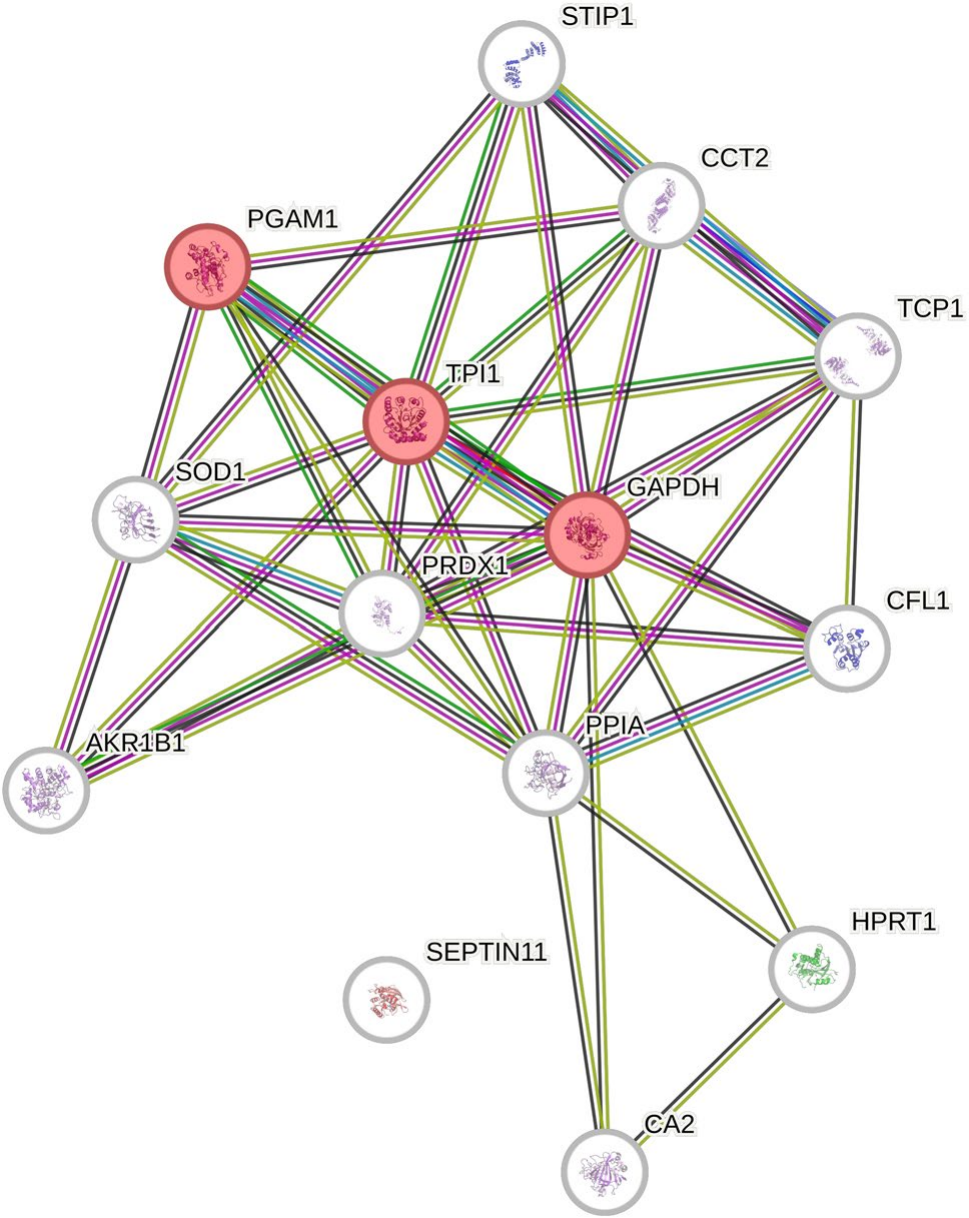
Ontological analysis of proteins shown to be differentially expressed in response to DENV 2 infection. STRING analysis of 14 host cell proteins shown to be differentially expressed after DENV 2 infection for 48 h as compared to mock infection. Proteins identified as involved in the glycolysis/gluconeogenesis pathway are represented in red.

STRING analysis of the four proteins identified at 12 h post infection (Stress-induced-phosphoprotein 1, threonine–tRNA ligase, glutamine-fructose-6-phosphate aminotransferase [isomerizing] 1 and glutathione S-transferase P) showed no linkage between the proteins, and there was no enrichment, shown by a PPI enrichment value of 1. STRING analysis (Fig. 2) of the proteins identified at 48 h post infection as differentially expressed in response to infection highlighted five KEGG pathways (Supplemental Table S3) including glycolysis/glucogenesis (3 proteins; false discovery rate 4.8E-03), biosynthesis of amino acids (3 proteins; false discovery rate 4.8E-03), carbon metabolism (3 proteins; false discovery rate 8.7E-03), fructose and mannose metabolism (2 proteins; false discovery rate 2.21E-02) and metabolic pathways (6 proteins; false discovery rate 2.21E^−02^). The PPI enrichment *p*-value was a highly significant < 1.0e^−16^, strongly suggesting that the proteins are, as a group, biologically connected. The KEGG pathway “metabolic pathways” was the most significantly enriched, containing six proteins, namely triosephosphate isomerase isoform 1, glyceraldehyde-3-phosphate dehydrogenase, aldo–keto reductase family 1 member B1, septin-11, carbonic anhydrase 2 and hypoxanthine–guanine phosphoribosyltransferase, However, when looking at the strength of the enrichment, the highest value was for fructose and mannose metabolism (strength 1.94, 2 out of 32)) closely followed by glycolysis/gluconeogenesis (strength 1.82, 3 out of 64). The metabolic pathways had the lowest strength value of 0.77 (6 out of 1435). It is notable that only one protein (STP1) was detected as differentially regulated at both time points (Tables 1 and 2).

### Validation of differentially expressed host proteins after DENV 2 infection

**Figure 3:**
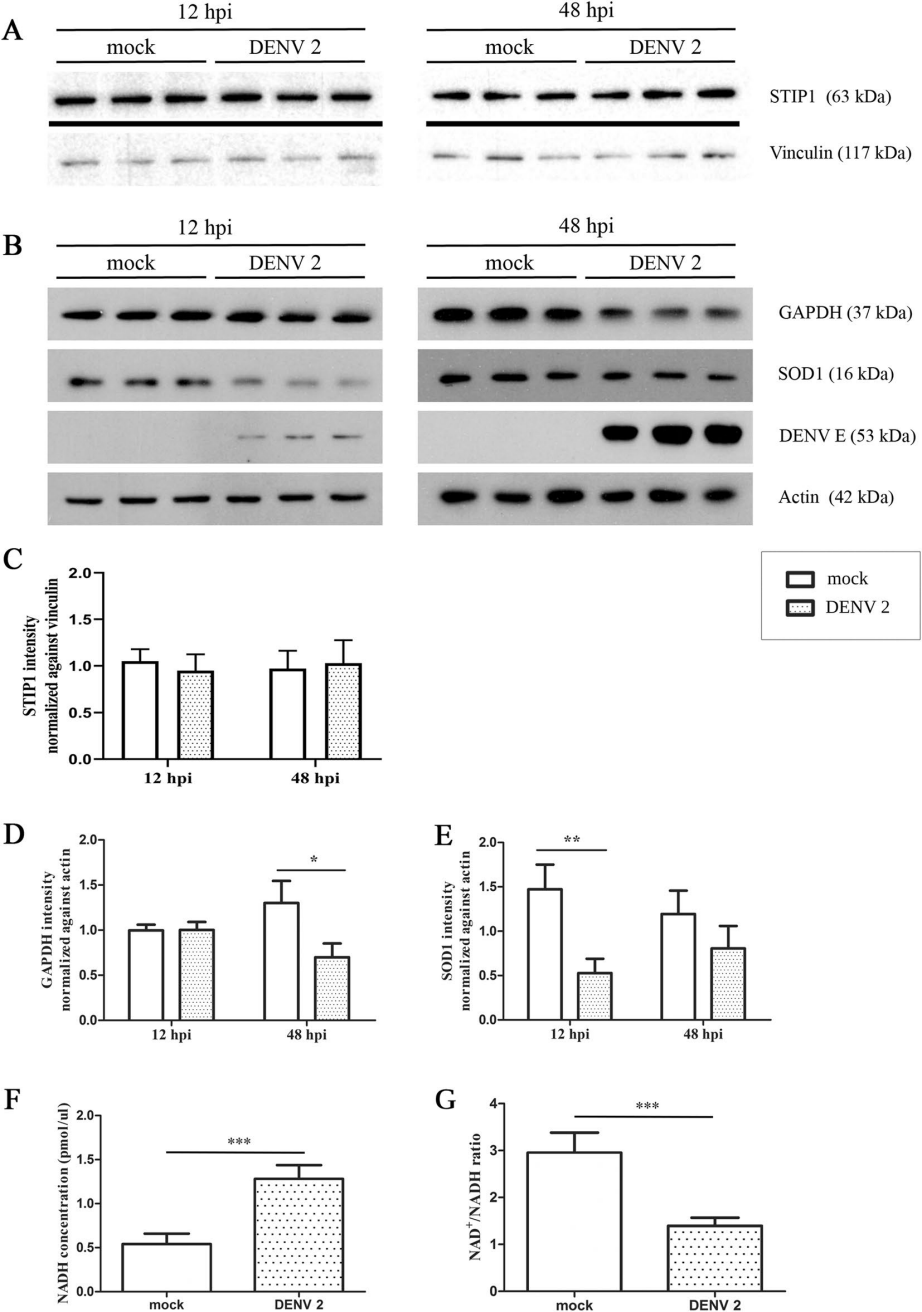
Validation of identified differentially expressed host proteins after DENV 2 infection in Hep3B cells. (**A)** Western blot of expression of STIP1 and Vinculin, (**B**) Western blot of expression of GAPDH, SOD1, DENV E protein and actin. (**C**, **D**) quantification of signal from western blots for (**C**) STIP1, (**D**) GAPDH and (**E**) SOD1. (**F**) ratio of NAD + /NADH and (**G**) level of NADH. *P* value < 0.05*, *P* value < 0.01** Composite images are shown consisting of successive antibody probings of the same membrane which are separated by (**A**) black lines, or (**B**) white bars. Full, uncropped western blots can be found in the supplemental materials.

Three proteins (STIP1, GAPDH and SOD1) were selected for validation. Hep3B cells were infected with DENV 2 and proteins extracted at 12 and 48 h and subsequently used in western blot analysis. Results showed that although the proteomic analysis had shown STIP1 as differentially expressed at both time points, western blot analysis did not confirm this, showing no difference in expression between mock infection and DENV 2 infection at both time points (Fig. 3A,C). The second candidate, GAPDH was significantly downregulated at 48 h post infection in agreement with the mass spectroscopy data (Fig. 3B, D and Table 1). The third candidate (SOD1) was however discordant, showing significant down regulation at 12 h post infection (that was not detected by the 2D analysis), but was reduced, albeit not significantly at 48 h (Fig. 3B,E).

### Analysis of GAPDH mRNA expression

**Figure 4:**
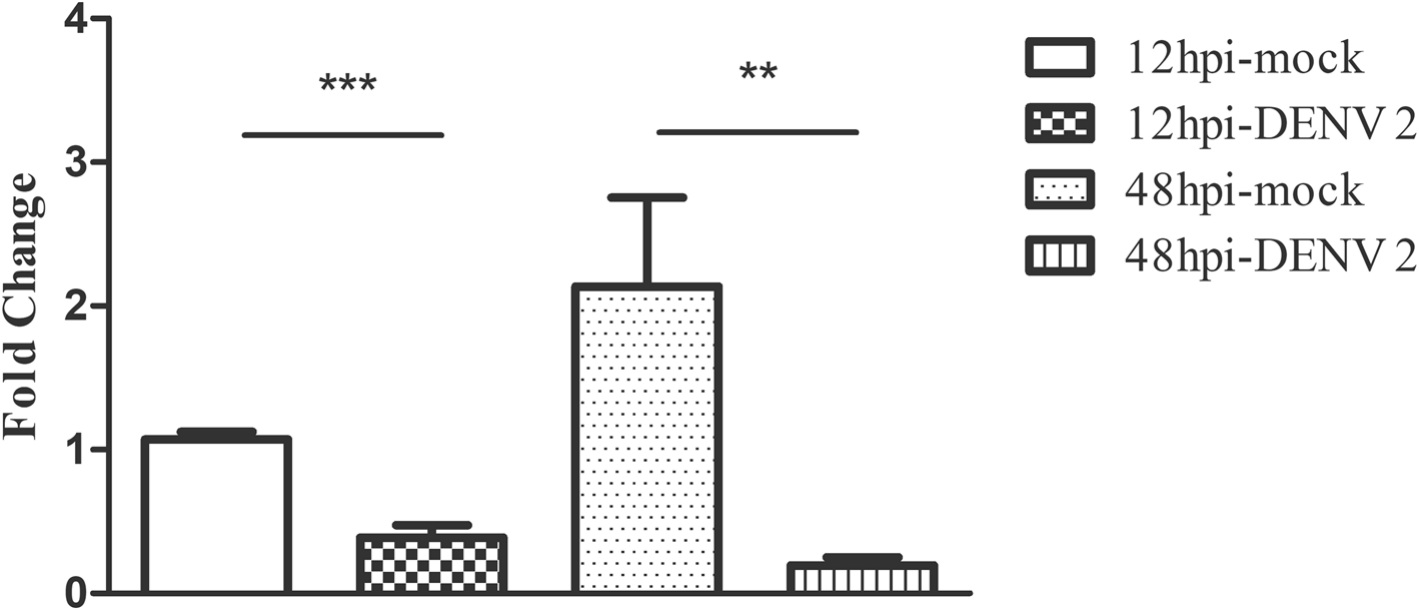
Expression of GAPDH mRNA. Hep3B cells were either mock infected or infected with DENV 2 for either 12 or 48 h. The experiment was undertaken as three independent biological replicates. The cell pellets were collected at the appropriate time and mRNA extracted and after conversion of cDNA the expression of GAPDH was quantitative real-time PCR. **P value < 0.01 ***P value < 0.001.

To determine whether the down-regulation of GAPDH was mediated at the level of transcription, or by a post-translational mechanism we investigated the mRNA level of GAPDH at 12 and 48 h.p.i, as compared to mock controls. The results (Fig. 4) showed that GAPDH expression was significantly down-regulated at both 12 and 24 h.p.i, showing that down-regulation of GAPDH at the protein level was mediated by a reduction in gene expression.

### Effect of DENV 2 infection on levels of NADH and NAD + 

To complete glycolysis to provide cellular energy, NAD + is required to donate electrons as part of the reaction, and NAD + exists as part of a reversible reaction with NADH. Excess NADH will serve to inhibit glycolysis as less NAD + is available. We therefore mock infected or infected Hep3B cells with DENV 2, and at 48 h post infection determined NADH levels and the NAD + /NADH ratio. The results (Fig. 3) showed that in response to infection NADH levels were significantly increased (Fig. 3F), and the NAD + /NADH ratio was significantly decreased (Fig. 3G), consistent with reduced glycolysis.

### Validation of GAPDH expression after DENV 2 infection in human hepatocyte-like cells

**Figure 5:**
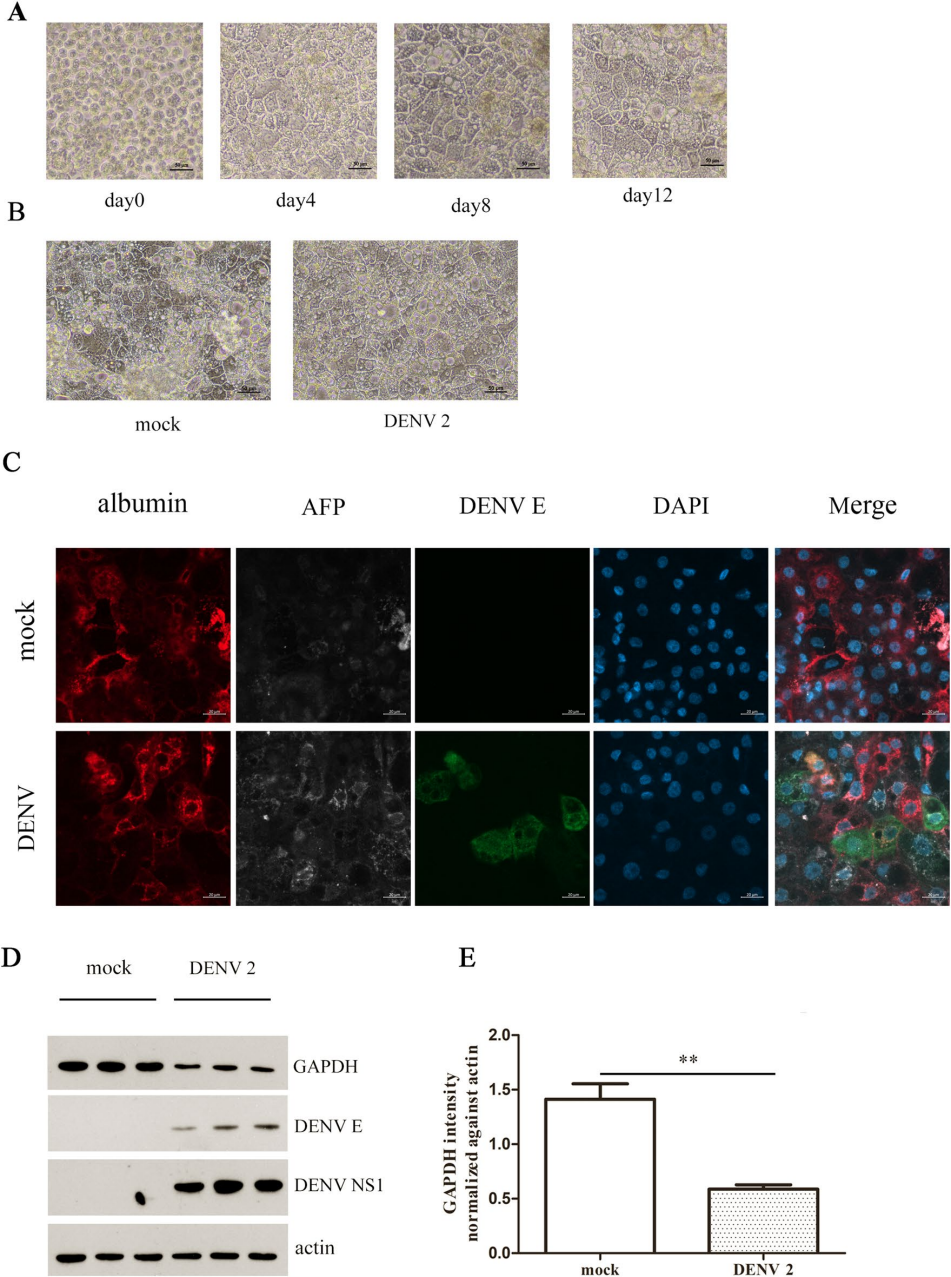
Validation of GAPDH expression in iPSC derived human hepatocytes. (**A**) Cryopreserved iPSC derived human hepatocytes were thawed and cultured with observation under a light microscope on days 0,4, 8 and 12. (**B**) When expressing mature hepatocyte markers (albumin and AFP) cells were either mock infected or infected with DENV 2 for 48 h and were observed under a light microscope. (**C**) Cells were subjected to immunofluorescence analysis using antibodies directed against albumin (RED), AFP (WHITE), DENV E (GREEN) and appropriate secondary antibodies after which cells were counterstained with DAPI (BLUE). (**D**) Mock infected and DENV 2 infected iPSC derived human hepatocytes at 48 hpi were collected, proteins prepared and used in western blot analysis with antibodies against GAPDH, DENV E protein, DENV NS1 and actin. (**E**) Quantification of GAPDH signal from (**D**). Experiments were undertaken as three independent biological replicates. *P* value < 0.01**Panel D is a composite image where successive antibody probings of the same membrane are separated by white bars. Full, uncropped western blots can be found in the supplemental materials.

Both 2D-PAGE and western blotting showed that GAPDH was decreased after DENV 2 infection in Hep3B cells which is a human hepatocarcinoma cell line. However, while cell lines can have properties which can resemble the original parental cell type, but the processes of immortalization and transformation can mean that the cell line can have extra or missing properties. We therefore further evaluated the expression of GAPDH in human hepatocyte-like cells to confirm the effect of DENV 2 to GAPDH in a more bona fide cell type. iPSC derived human hepatocytes were cultured (Fig. 5A) and when they expressed mature hepatocyte markers were either mock infected or infected with DENV 2 at MOI 5 (Fig. 5B). After 48 h the expression of the mature hepatocyte markers albumin and alpha-fetoprotein (AFP) were re-confirmed as well as expression of DENV E protein evaluated in both mock—and DENV 2 in iPSC derived human hepatocytes (Fig. 5C) by immunohistochemistry and confocal microscopy. In parallel proteins were extracted from the cells, and the expression of GAPDH determined by western blotting. The results (Fig. 5D,E) showed that GAPDH was significantly down-regulated in DENV 2 infected cells when compared to mock infected cells which is consistent with the results seen with DENV 2 infected Hep3B infected cells.

### Effects of metformin on DENV 2 infection in Hep3B cells

**Figure 6:**
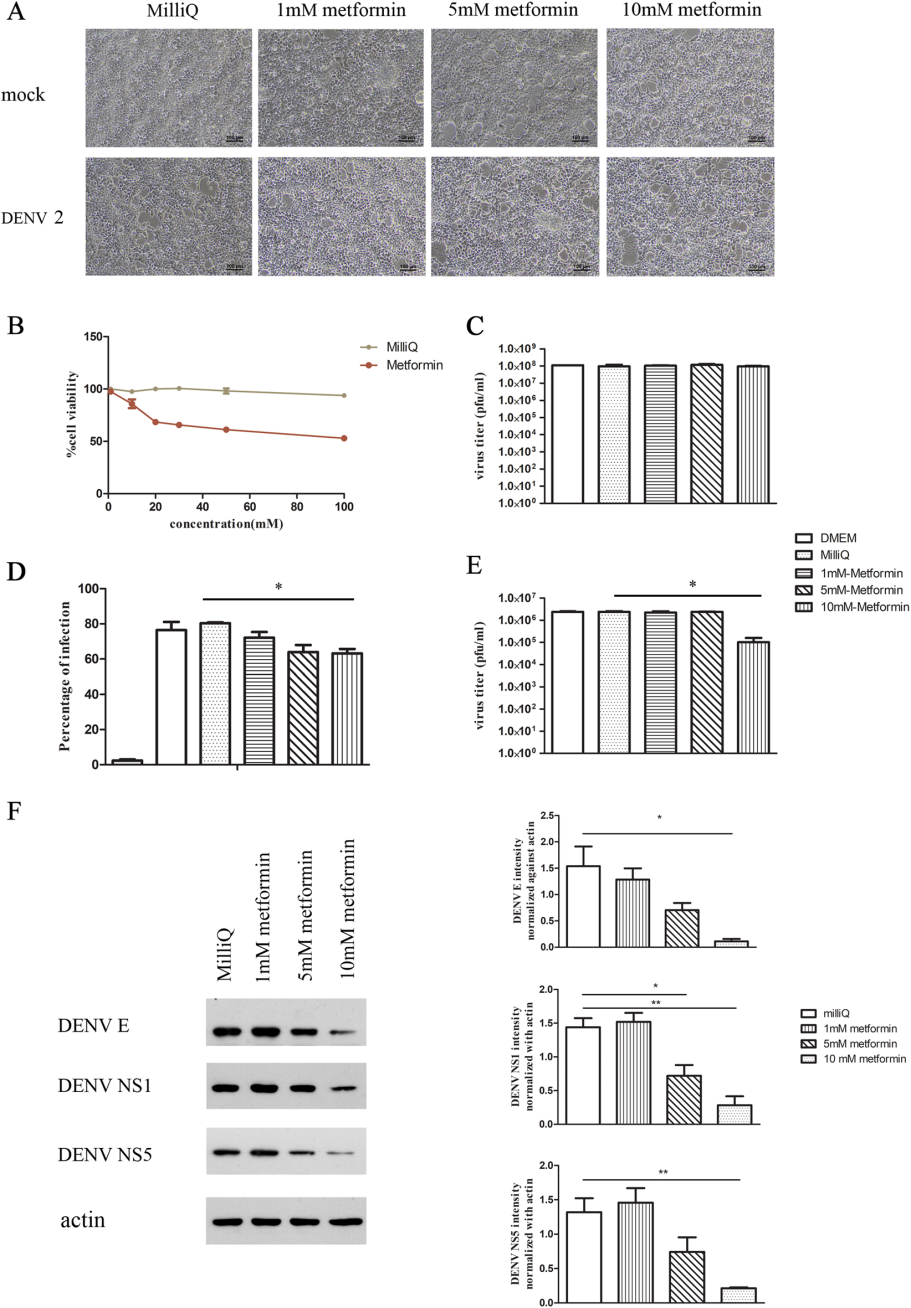
Effects of metformin on DENV 2 infected Hep3B cells. Hep3B cells were treated with various concentrations of metformin and (**A**) observed under a light microscope to see cell morphology and (**B**) after treatment for 24 h cytotoxicity was determined by the MTT assay. (**C**) Stock DENV 2 was incubated with various concentrations of metformin for 1 h at 37 °C after which titer was evaluated by plaque assay. DENV 2 infected Hep3B cells were treated with various concentrations of metformin for 24 h after which (**D**) level of DENV infection was determined by flow cytometry and (**E**) level of virus in the supernatant determined by plaque assay. (**F**) Proteins from metformin treated or vehicle treated DENV 2 infected Hep3B cells were prepared and used in western blot analysis to determine expression of DENV E protein, DENV NS1 protein, DENV NS5 protein and actin, signals were quantitated as shown. Experiments were undertaken as three independent biological replicates. *P* value < 0.05*, < 0.01** Panel F contains a composite image where successive antibody probings of the same membrane are separated by white bars. Full, uncropped western blots can be found in the supplemental materials.

Metformin is a widely used drug to treat diabetes mellitus that acts by inhibiting hepatic gluconeogenesis^28^. To investigate how this drug affects DENV 2 infection, we first established the cytotoxicity of this drug towards HepG2 cells using the MTT assay. The results (Fig. 6A,B) showed minimal cytotoxicity at concentrations lower than 10 mM metformin, but some cytotoxicity above this concentration. The calculated 50% cytotoxic concentration (CC_50_) was 104.34 mM (Fig. 6B). We subsequently determined whether metformin has detrimental effects on the virus directly, undertaking a virucidal assay in which stock DENV 2 with a known titer was incubated directly with different concentrations of metformin or control vehicle for 1 h at 37 °C, before determining the solution titer by plaque assay. The results showed that metformin had no virucidal effect on DENV 2 (Fig. 6C). To determine the effect of metformin in infection, Hep3B cells were mock infected or infected with DENV and after viral adsorption, 1, 5 or 10 mM metformin diluted in milliQ were added to the cells. At 24 h.p.i, cells and supernatant were collected. We observed that the both dengue viral protein and infectious virion were significantly reduced in a dose-dependent manner after post-infection treatment with metformin for 24 h (Fig. 6D–F). The calculated EC_50_ for metformin on virus production was 6.77 mM, giving a selectivity index (SI) of 15.41, supportive of true antiviral activity. These results suggest that metformin may possesses anti-DENV activity through metabolism inhibition of gluconeogenesis, one of the two pathways (together with glycogenesis) essential for glucose homeostasis and energy production.

Lastly, we sought to investigate the effect that metformin was having on GAPDH directly. We selected the same time point as the other drug treatment experiments (24 h.p.i). Cells were then either mock infected, treated with 10 mM metformin, infected with DENV 2 alone, or infected with DENV 2 and treated with 10 mM metformin. The expression of GAPDH was then determined at the level of the mRNA and at the level of the protein. In addition, the NADH concentration and NAD + /NADH ratio were determined. The results (Supplementary Fig. S8A) showed that metformin treatment increase mRNA expression of GAPDH over mock infection, and surprisingly DENV 2/metformin increased GAPDH over infection alone. However, at the level of the protein no differences in expression were seen between any treatment including increasing amounts of metformin in mock infected cells (Supplementary Fig. S8B) or in DENV 2 infected cells (Supplementary Fig. S8C). Metformin treatment alone reduced the concentration of NADH as compared to untreated cells to a level that was similar to the levels seen with DENV 2 infection and DENV 2/metformin treatment (Supplementary Fig. S8D). Lastly, no differences were seen in NAD + /NADH ratio between any of the treatments (Supplementary Fig. S8E).

## Discussion

A number of cellular targets of DENV have been identified including immune cells^29^, various blood cells^30,31^ and hepatocytes^7^. To establish a successful infection, DENV must overcome the host innate immunity as well as re-engineer the host cell to produce an environment that facilitates and promoted viral replication. This re-engineering is achieved by a small complement of only 10 proteins and the genomic material itself. The re-engineering of the host cell is hallmarked by changes in protein expression and in lipid composition of the host cell.

Despite the importance of the liver and hepatocytes as a target organ only a few studies have investigated the protein changes that occur in DENV infection of hepatocytes^19–21^. To address this issue we initially investigated the host proteomic changing occurring in DENV 2 infected human liver cancer (Hep3B) cells. It should be noted that our study was undertaken using a culture buffer “mock infection” control, which while very widely used may have some limitations compared to the use of an inactivated virus control. Very few changes in protein expression were detected at 12 hpi, while a significantly greater number were detected at 24 hpi. Only one protein, STP1, was detected at both time points investigated, and this protein failed during validation, and no difference in expression between mock infected and DENV 2 infected cells was seen at either time point (12 and 48 hpi). Similarly, SOD1 which was identified as differentially expressed (downregulated in response to infection) at 48 hpi in the proteome analysis was found to be reduced, but not significantly at 48 hpi in western blot analysis, but was significantly down regulated in DENV 2 infected cells at 12 hpi, albeit that this was not detected in the original proteome analysis.

GAPDH was one of the three glycolysis/gluconeogenesis proteins detected, and was shown to be significantly down regulated at 48 hpi. GAPDH catalyzes the sixth step of glycolysis and catalyzes the conversion of glyceraldehyde 3-phosphate to D-glycerate 1,3-bisphosphate. Previous studies on the role of glycolysis in DENV infection have been somewhat contradictory. Fontaine and colleagues^32^ showed that DENV infection both induced and requires glycolysis for optimal viral replication, and it was proposed that the enhancement of glycolysis was required to support the metabolic requirements for DENV replication. Allonso and colleagues^33^ proposed a mechanism mediating the increased glycolysis by reporting that DENV NS1 protein directly interacts with GAPDH and increases glycolytic flux. Indeed, they noted that the expression of DENV NS1 protein alone could increase glycolytic flux ^33^. However, these results are sharply contrasted by Silva and colleagues^34^ who showed that DENV NS3 protein interacted with GAPDH and reduced glycolytic activity. This latter study would correspond with this study, showing a decrease in GAPDH expression, and a decrease in glycolytic flux as indicated by a significant increase in NADH levels and a significant decrease in the NAD + /NADH ratio. As proposed by Silva and colleagues^34^ it is possible that DENV infection may shift the cellular metabolism to alternate, non-glycolytic pathways. It is also important to note that in our study, the reduction of GAPDH protein levels in DENV infection was confirmed in iPSC derived hepatocytes, strongly supporting our results. Indeed, while our study and the study by Silva and colleagues^34^ were undertaken using liver cells, while Fontaine and colleagues investigated primary human foreskin fibroblasts and human telomerase reverse transcriptase-immortalized microvascular endothelial (TIME) cells^32^. The remaining study^33^ used baby hamster kidney fibroblasts (BHK-21 cells) and human umbilical vein endothelial cells (HUVEC-C cells). Thus, it might not be coincidental that the studies undertaken in liver cells are concordant, while differing from studies undertaken cell types.

Gluconeogenesis is the process by which glucose is produced from non-carbohydrate precursors which is then fed into the glycolysis pathway, and metformin has long been used as an inhibiting agent of this pathway in treatment of type 2 diabetes mellitus (T2DM) through its effect on hepatic gluconeogenesis, and recently a protocol regarding the use of metformin as a therapy for dengue in overweight and obese patients has been published^35^, although the results of this study have not yet been published. Interestingly, metformin significantly reduced both the level of DENV infection, and the level of viral output when added to infected DENV cells, a result consistent with a previous study looking at the effects of metformin on DENV, ZIKV and YFV infection in Huh-7 (liver) cells^36^. This would argue that, given the reduction of glycolysis, glucose is still necessary, but is being used in alternate pathways such as the pentose phosphate pathway to generate nucleotides^37^ that are also heavily required during viral replication.

It should be noted that one study has proposed that metformin is not suitable as an antiviral agent. The authors found that metformin had poor antiviral activity against all four DENV serotypes in BHK-21 cells, and that metformin was actually pro-viral in Vero cells^38^. However again it needs to be emphasized that studies undertaken in liver cells tend to be concordant, but can often be discordant to results generated in other cell types.

Efforts to further probe the interaction between metformin treatment and GAPDH were relatively unsuccessful. The experiments were undertaken at a time point consistent with the previous metformin experiments (24 h.p.i), rather than at the time point consistent with the proteome and western blot analyses (48 h.p.i). This probably sheds light on the infection process, with it requiring at least 48 h post infection to see changes in GAPDH expression. This might suggest that normal glycolysis is required initially in the infection process, with modulated expression being required at a later time point. Future studies may need to address this question more fully.

It has to be noted that other studies, including our own, have regularly used GAPDH as a western blot loading control without observing any significant down regulation as seen here. At least two of our previous studies^39,40^ used GAPDH as a loading control without observing down regulation. While they used the same virus (DENV 2; strain 16681), the previous studies were conducted in HepG2 cells while this study was conducted in Hep 3B cells. Similarly, the study of Jianshe and colleagues^41^ also used human iPSC generated hepatocyte-like cells to investigate DENV infection and saw no GAPDH down-regulation. However, the strains of DENVs used were different from DENV 2 (16681). Collectively these results point to both virus strain and cell type in being variables that can modulate the results. In a recent study we showed that two Zika virus strains differing by only 9 amino acid changes interacted in markedly distinct way with the host cell miRNA machinery^42^. We previously published an analysis of the protein changes induced by 12 DENVs of different serotypes and origin (DENV 1–4; low passage isolates from dengue fever, low passage isolates from dengue hemorrhagic fever and laboratory adapted isolates). A peptidome analysis showed that only 20% of peptides were expressed in all samples, while 80% showed variable expression. As a result of this we suggested the term “plasticity” in the dengue virus host cell interaction, and proposed that similar over all outcomes could be achieve in different ways^43^. For example, it is possible that some strains of DENV inhibit glycolysis through enzymatic inhibition^34^, while others may obtain the same objective through down-regulation of the level of GAPDH mRNA and protein (as seen here). Overall, the results suggest that although GAPDH is widely used as a loading control, it is not a bona fide housekeeping gene, and its use and interpretation should be carefully monitored in viral infection studies. Further studies investigating the role of GAPDH in DENV infection may well be difficult, especially given the apparent cell line/virus strain effects. In addition, the central role of GAPDH in not only glycolysis, but also in other processes such as autophagy^44^, a process known to play a critical role in DENV infection^39,45^, will make evaluating the role of GAPDH technically difficult.

While it is clear that increased energy is required for DENV replication, it is unclear how this is obtained. Indeed, Heaton and Randal proposed that it is DENV induced autophagy that modulates lipid metabolism to increase cellular β-oxidation and thus increases generation of ATP^46^. They additionally proposed that DENV NS3 protein interacted with fatty acid synthase to redistribute it to viral replication sites, thus increasing cellular fatty acid synthesis^47^. Thus, the overall mechanism of how energy is generated to support viral replication remains to be elucidated, but it is possible that inhibition of the glycolysis pathway dives increased production of nucleotides through the pentose phosphate pathway, while alterations in lipid metabolism compensate for shortfalls in energy derived through the process of glycolysis and the TCA cycle.

Our previous proteomic analysis of DENV infection of liver cells focused on the mechanism of action of andrographolide in DENV infection^19^. In that study, andrographolide had a very minor effect on GAPDH, down-regulating it slightly. However, the combination of DENV infection and andrographolide treatment produced a significant down regulation of GAPDH. However, in that study GAPDH was not down regulated by DENV infection alone, but it should be noted that the previous work was undertaken at 24 hpi, while this study was undertaken at 48 hpi. The lack of down regulation of GAPDH by DENV 2 at 24 h.p.i. is thus consistent between this study and the prior study. The study by Pattanakitsakul and colleagues^21^ identified 17 proteins as differentially regulated in response to DENV infection of HepG2 liver cells, none of which are consistent with the proteins detected in this study, but again that study was performed at 24 hpi. The study by Pando-Robles and colleagues^20^ used a high throughput methodology (label free LC–MS) and identified a total of 155 differentially expressed proteins in response to DENV infection of Huh-7 liver cells, of which 64 were upregulated, and 91 were downregulated. GAPDH was identified as a moderately upregulated protein, but other significantly downregulated protein in glycolysis and the TCA cycle led the authors to conclude that DENV infection reduces energy metabolism, consistent with the results presented here and those of Silva and colleagues^34^.

## Conclusion

Despite the importance of the liver as a target of DENV infection, little is known as to the global proteome changes undergone in this cell type upon infection. Indeed, only three studies have previously undertaken proteomic analysis of DENV infected liver cells^19–21^. One protein (GAPDH) that was detected in the 2D-proteome analysis of DENV 2 infected Hep3B cells as down regulated was validated in western blot of Hep3B cells, as well as, importantly, in iPSC derived human hepatocytes. Consistent with the results, NADH levels were significantly increased, and the NAD + /NADH ratio was significantly decreased, consistent with reduced glycolysis. Metformin, an inhibitor of hepatic gluconeogenesis, significantly reduced the level of cellular infection and additionally new virus production. Collectively these results suggest that glycolysis is reduced in liver cells during DENV infection with glucose being utilized for nucleoside production through the pentose pathway. It is likely that the energy deficit is compensated for by increased β-oxidation generated through increased lipid metabolism, as suggested by others^46^.

### Supplementary Information

Supplementary Information.

## Data Availability

All data generated or analyzed during this study are included in this published article (and its Supplementary Information file).
