# Supplementary material for: Glycolysis is reduced in dengue virus 2 infected liver cells
Source: Sci Rep. 2024 Apr 9;14:8355. doi: 10.1038/s41598-024-58834-w (PMC11004007; doi:10.1038/s41598-024-58834-w)

Supplementary materials

Glycolysis is reduced in dengue virus 2 infected liver cells.

Chanida Chumchanchira[1,2], Suwipa Ramphan[2], Wannapa Sornjai[2], Sittiruk Roytrakul[3], Pathrapol Lithanatudom[4*], Duncan R. Smith[2*]

[1]PhD Degree Program in Biology, Faculty of Science, Chiang Mai University, Chiang Mai, 50200, Thailand

[2]Institute of Molecular Biosciences, Mahidol University, Nakhon Pathom, 73170, Thailand.

[3]National Center for Genetic Engineering and Biotechnology (BIOTEC), National Science and Technology Development Agency, Pathum Thani, 12120, Thailand

[4]Department of Biology, Faculty of Science, Chiang Mai University, Chiang Mai, 50200, Thailand.

***Correspondence to:** Pathrapol Lithanatudom (pathrapol_li@hotmail.com) or Duncan R. Smith (duncan_r_smith@hotmail.com).

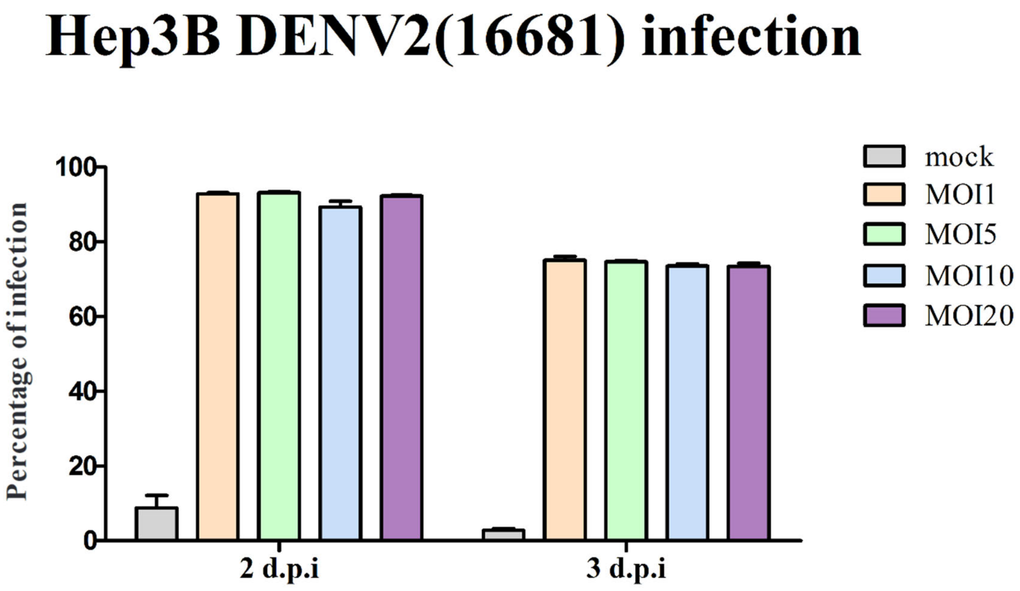

Supplemental Figure 1. Optimization of DENV 2 infection of Hep3B cells.

Hep3 cells were mock infected or infected with DENV 2 at different MOIs. At 48 and 72 hours post infection the level of infection was determined by flow cytometry.

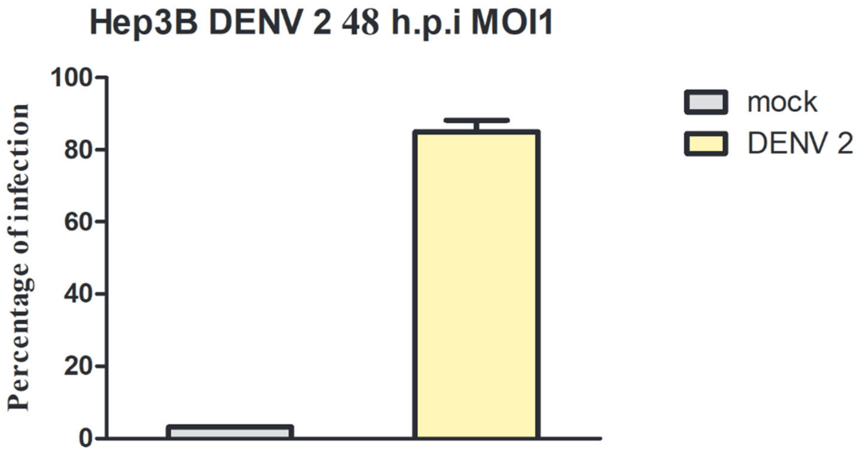

Supplemental Figure 2. Confirmation of optimized DENV infection protocol.

Hep3 cells were mock infected or infected with DENV 2 at an MOI of 1. At 48 hours post infection the level of infection was determined by flow cytometry.

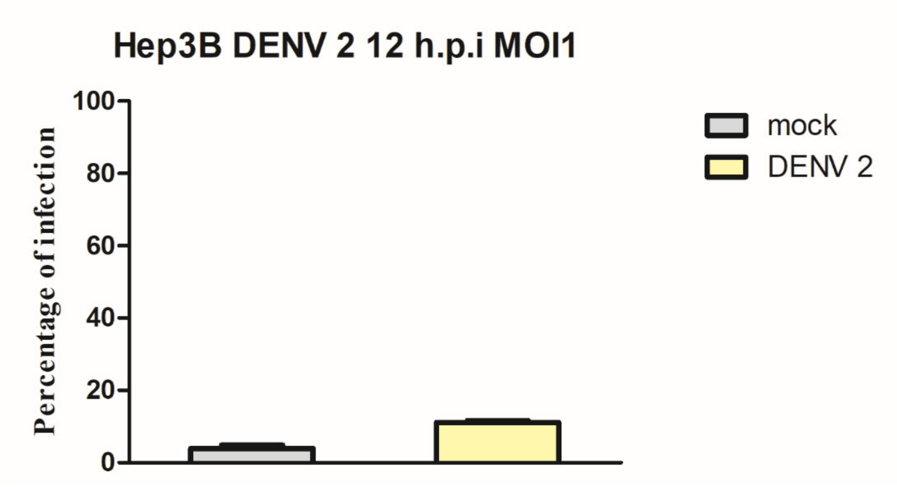

Supplemental Figure 3. Level of infection of Hep3B cells after DENV 2 infection for 12 hours.

Hep3 cells were mock infected or infected with DENV 2 at an MOI of 1. At 12 hours post infection the level of infection was determined by flow cytometry.

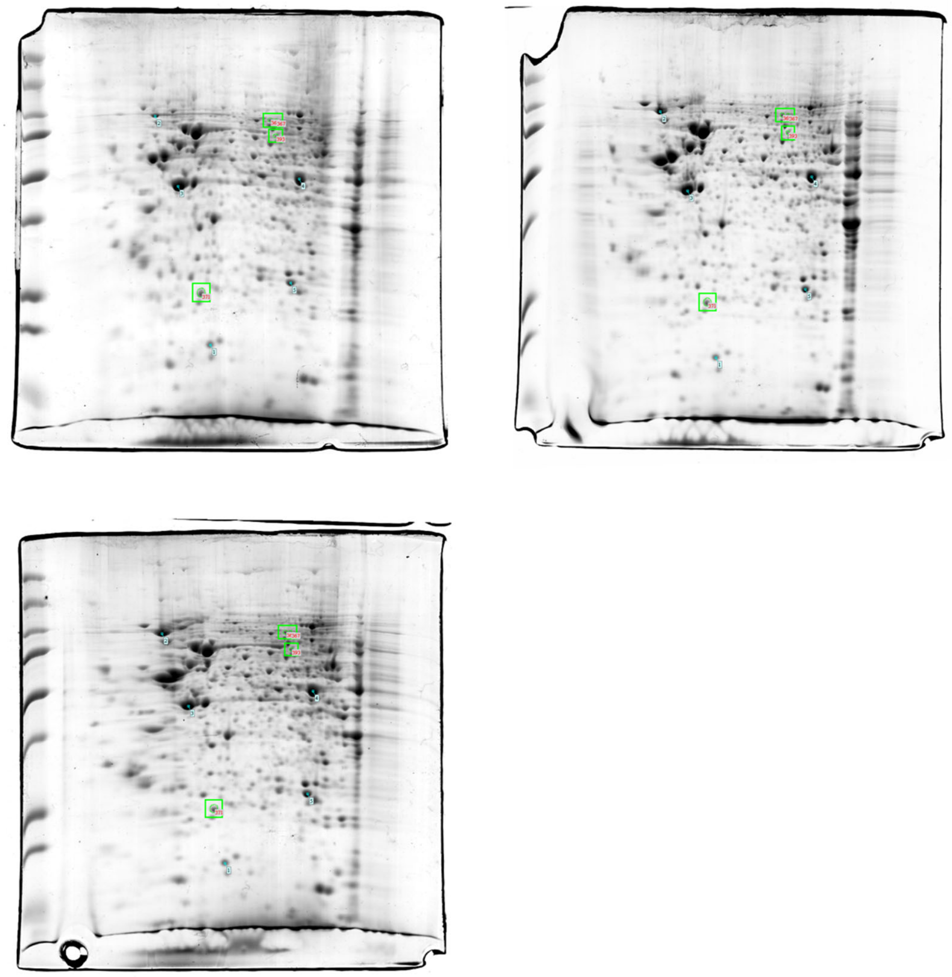

Supplemental Figure S4.

Three replicate 2-D gels of Hep3B cells after mock infection for 12 hours.

Replicate 1

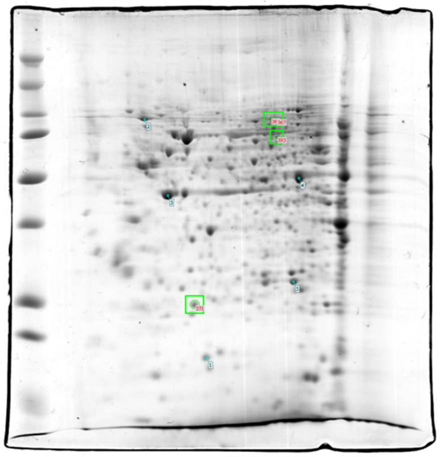

Replicate 2

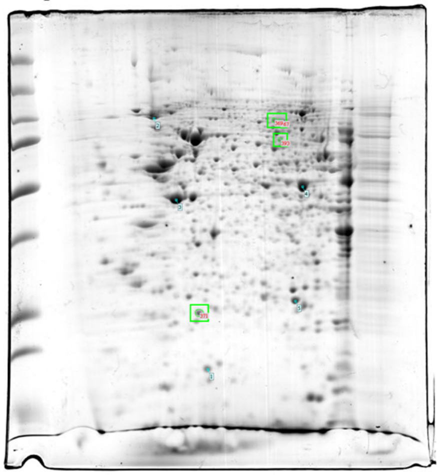

Replicate 3

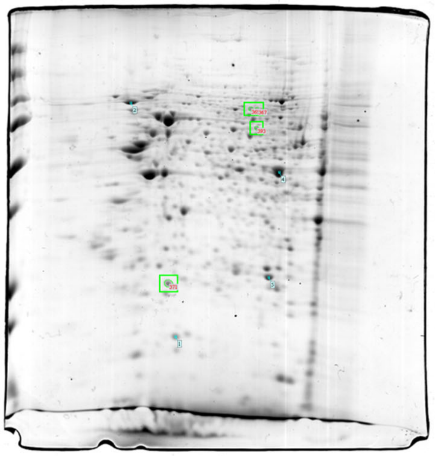

Supplemental Figure S5.

Three replicate 2-D gels of Hep3B cells after DENV 2 infection for 12 hours.

Replicate 1

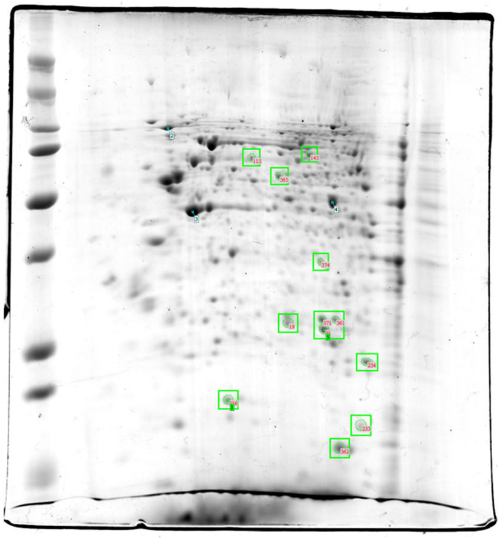

Replicate 2

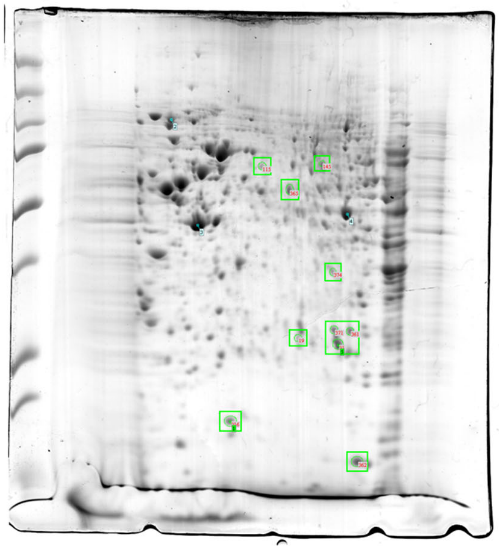

Replicate 3

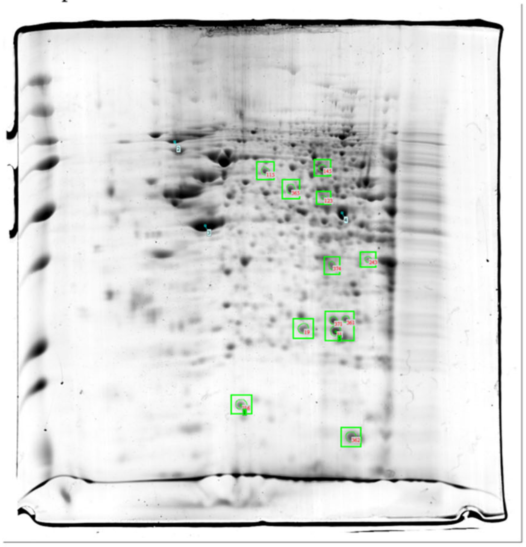

Supplemental Figure S6.

Three replicate 2-D gels of Hep3B cells after mock infection for 48 hours.

Replicate 1

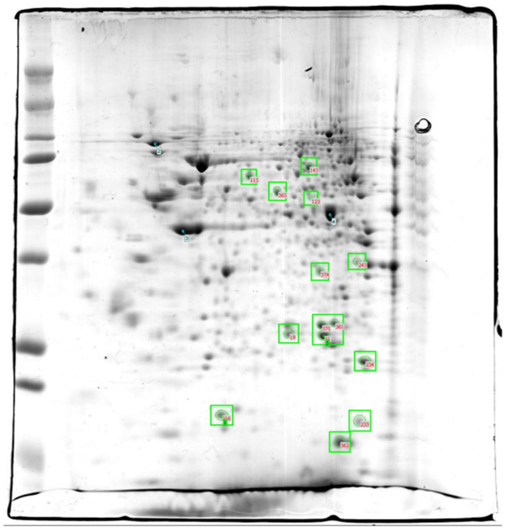

Replicate 2

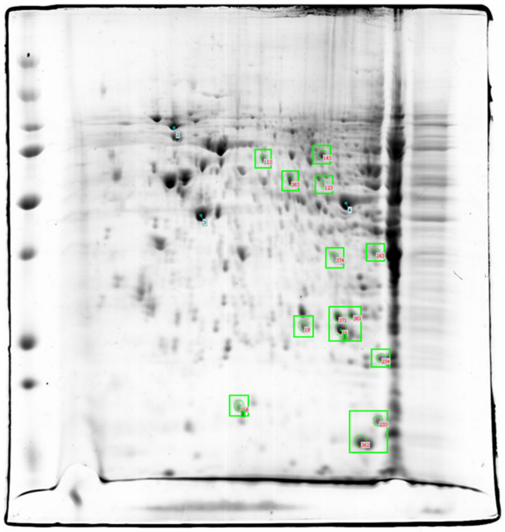

Replicate 3

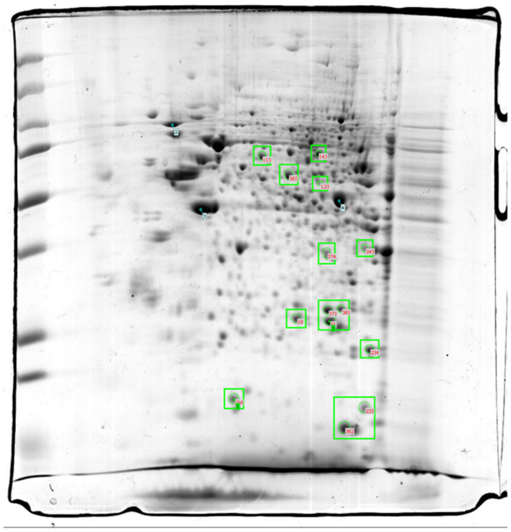

Supplemental Figure S7.

Three replicate 2-D gels of Hep3B cells after DENV 2 infection for 48 hours.

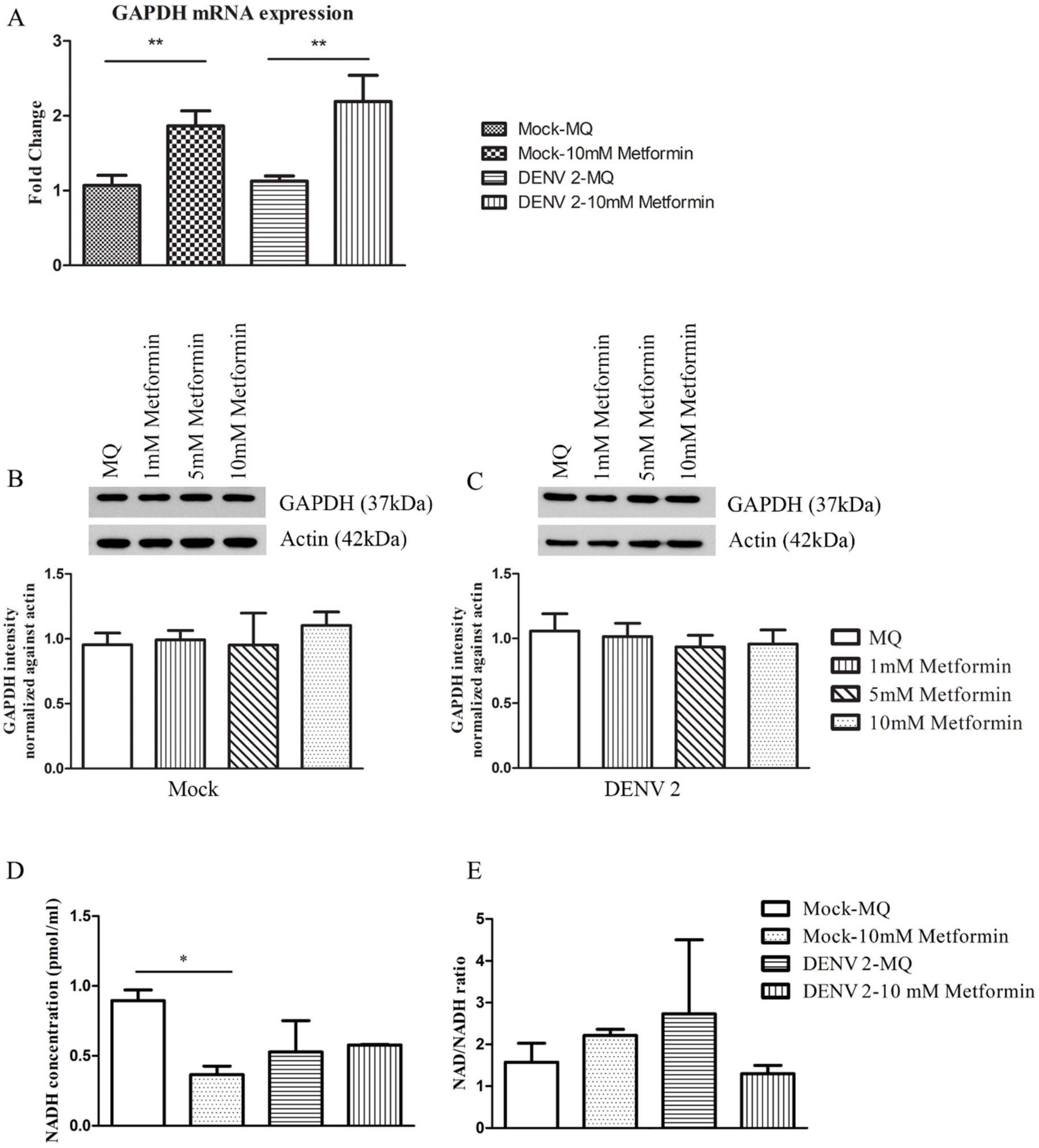

Supplemental Figure S8. GAPDH and NAD+/NADH response to metformin.
(A) Hep3B cells were either treated with Milli-Q water, or 10mM metformin, or infected with DENV 2, or treated with10 mM metformin and infected with DENV 2. At 24 h.p.i. RNA was extracted and the level of GAPDH mRNA determined by real-time quantitative PCR. Hep3B cells were either treated with Milli-Q water or with 1mM, 5mM or 10 mM metformin before being (B) mock infected or (C) infected with DENV 2. At 24 h.p.i. cells were harvested and proteins collected. Protein samples were used in a western blot to detect the levels of GAPDH and actin as a loading control. Hep3B cells were either treated with Milli-Q water, or 10mM metformin, or infected with DENV 2, or treated with10 mM metformin and infected with DENV 2. At 24 h.p.i. the NADH concentration and NAD+/NADH ratio determined.

Supplementary Table S1. Antibodies used in western blots

Name of antibody	Source	Type	Dilution	Detail
Anti-STIP1 (ab56873; Abcam plc, Cambridge, UK)	mouse	polyclonal	1:5000	1°Ab
Anti-SOD1 (ab56873; Abcam plc, Cambridge, UK)	rabbit	polyclonal	1:5000	1°Ab
Anti-GAPDH (6C5) (sc-32233; Santa Cruz, Biotechnology Inc., Texas, USA)	mouse	polyclonal	1:8000	1°Ab
Pan specific anti-flavivirus E protein (ATCC: HB-112)	mouse	monoclonal	1:500	1°Ab
Anit-NS5 (DENV-2)(PA5-278888; Pierce, Rockford, IL, USA)	rabbit	polyclonal	1:3000	1°Ab
Anti-NS1 (DENV-2)(PA5-278885; Pierce, Rockford, IL, USA)	rabbit	polyclonal	1:5000	1°Ab
Anti-Vinculin (N-19) (sc-7649; Santa Cruz, Biotechnology Inc., Texas, USA)	goat	polyclonal	1:5000	1°Ab
β-Actin (C4)-HRP (sc-4778HRP; Santa Cruz, Biotechnology Inc., Texas, USA)	mouse	monoclonal	1:20000	1°Ab
HRP-conjugated goat anti-mouse IgG (A4416; Sigma, MO, USA)	goat	polyclonal	1:5000	2°Ab
HRP-conjugated rabbit anti-goat IgG (31402; Pierce, IL, USA)	rabbit	polyclonal	1:5000	2°Ab
HRP-conjugated goat anti-rabbit IgG (31460; Pierce, IL, USA)	goat	polyclonal	1:5000	2°Ab

Supplementary Table S2. Antibodies used in immunofluorescence assay

Name of antibody	Source	Type	Dilution	Detail
Anti-Albumin (ab207327; Abcam plc, Cambridge, UK)	rabbit	polyclonal	1:50	1°Ab
Anti-Alpha fetoprotein (A85176; Antibodies, Davis, CA, USA)	goat	polyclonal	1:50	1°Ab
Pan specific anti-dengue virus type 1-4 antibody (MA1-27093; Pierce, Rockford, IL, USA)	mouse	polyclonal	1:100	1°Ab
Alexa Fluor® 488 donkey anti-mouse IgG antibody (A11029; Invitrogen)	donkey	polyclonal	1:100	2°Ab
Alexa Fluor® 568 donkey anti-goat IgG antibody (A11057; Invitrogen, Waltham, Massachusetts, USA)	donkey	polyclonal	1:100	2°Ab
Alexa Fluor® 647 donkey anti-rabbit IgG antibody (A31573, Invitrogen, Waltham, Massachusetts, USA)	donkey	polyclonal	1:100	2°Ab

Supplementary Table S3. Functional enrichments in KEGG pathways analyzed by the STRING bioinformatic analysis software.

**KEGG Pathways**			
Pathway ID	*Pathway discription*	*Count**	*FDR***
hsa00051	Fructose and mannose metabolism	2	0.0221
hsa00010	Glycolysis/Glucogenesis	3	0.0048
hsa01230	Biosynthesis of amino acids	3	0.0048
hsa01200	Carbon metabolism	3	0.0087
hsa01100	Metabolic pathways	6	0.0221

*Count: Count in gene set

**FDR: False discovery rate.

Uncropped figures

Fig 3A:
(STIP1)

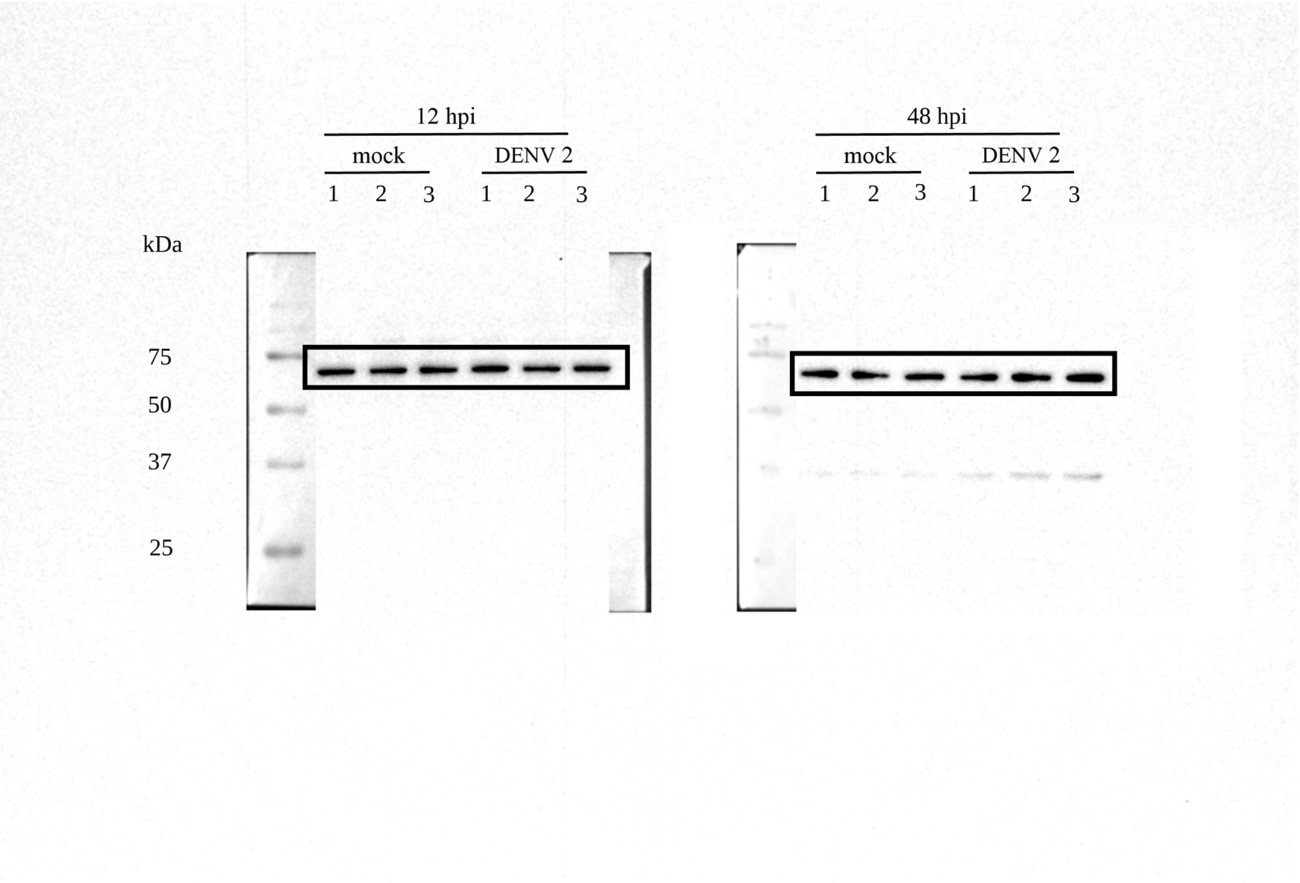

Fig 3A:
(vinculin)

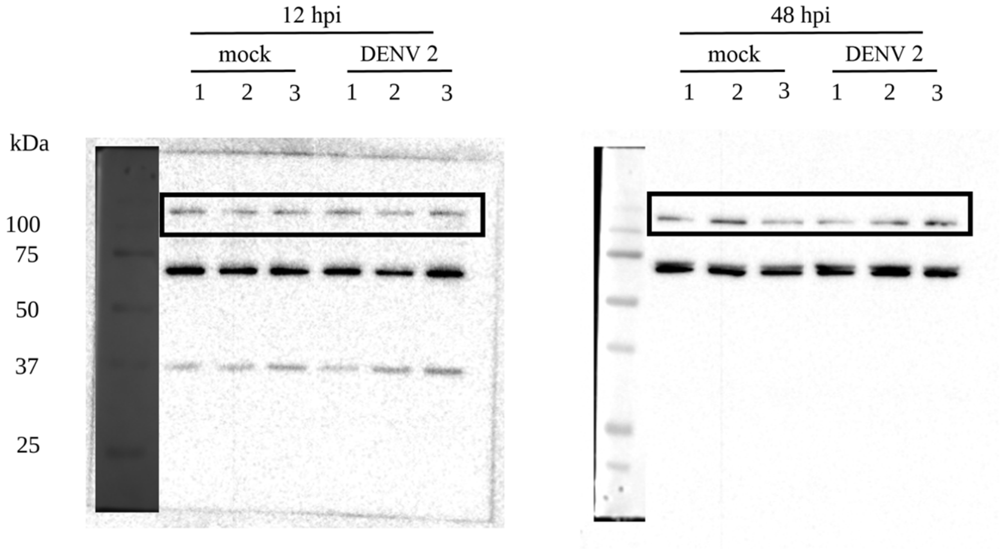

Fig 3B:
(GAPDH)

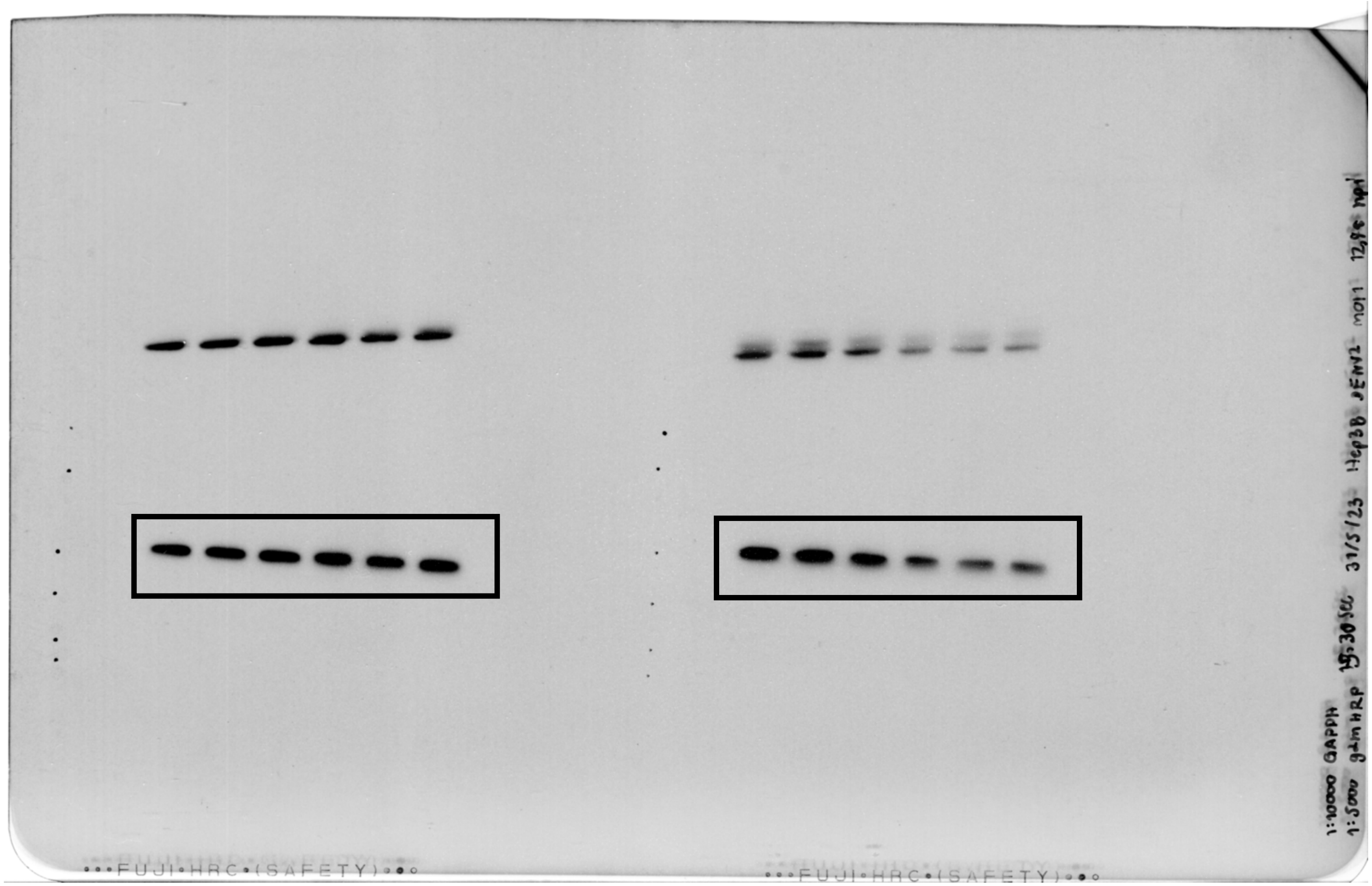

Fig 3B:
(SOD1)

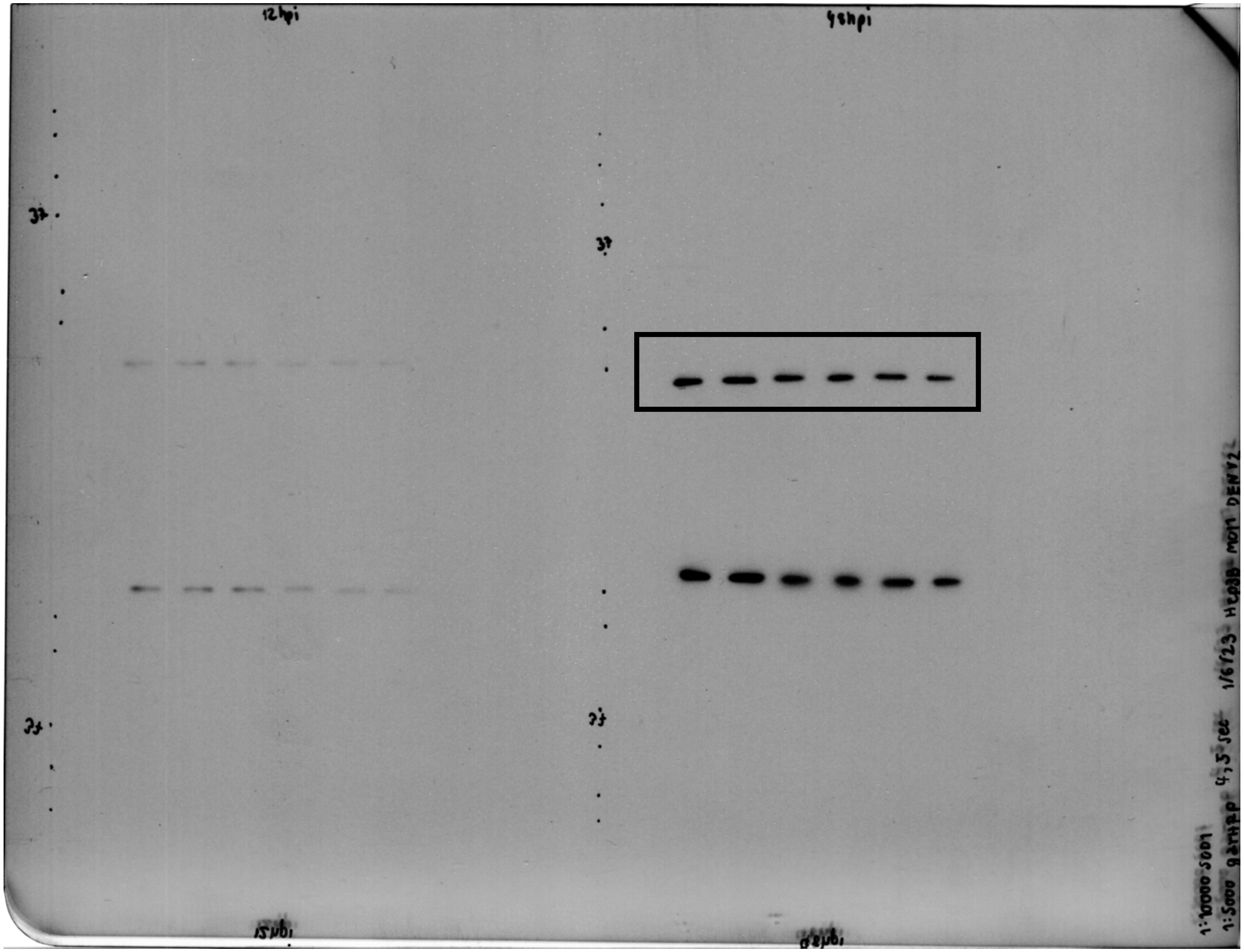

Fig 3B:
(SOD1)

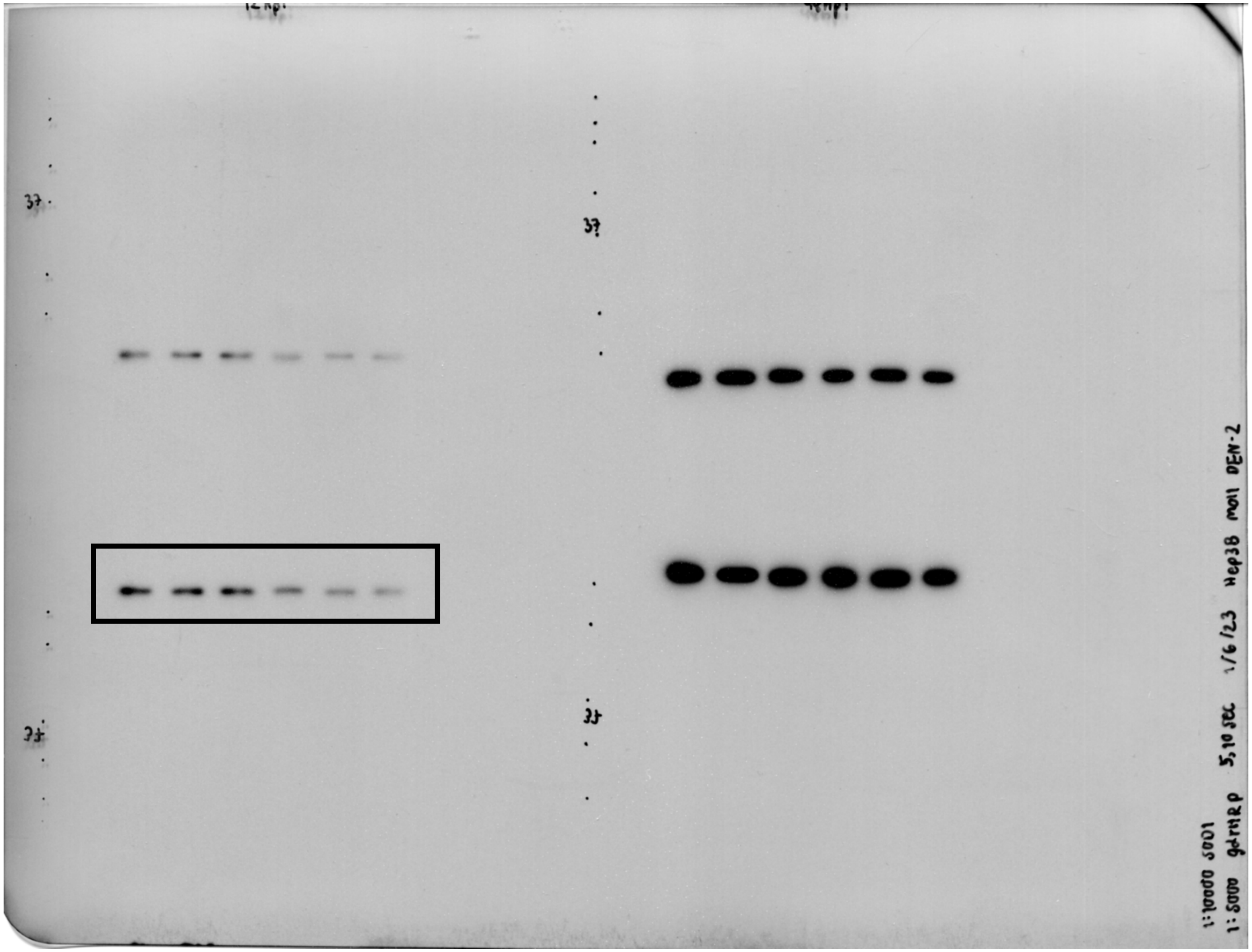
37
37
37
37
1:10000 SOD1
1:5000 gαrHRP 5,10 SEC 1/6/23 Hep3B moi1 DEN-2

Fig 3B:
(DENV E)

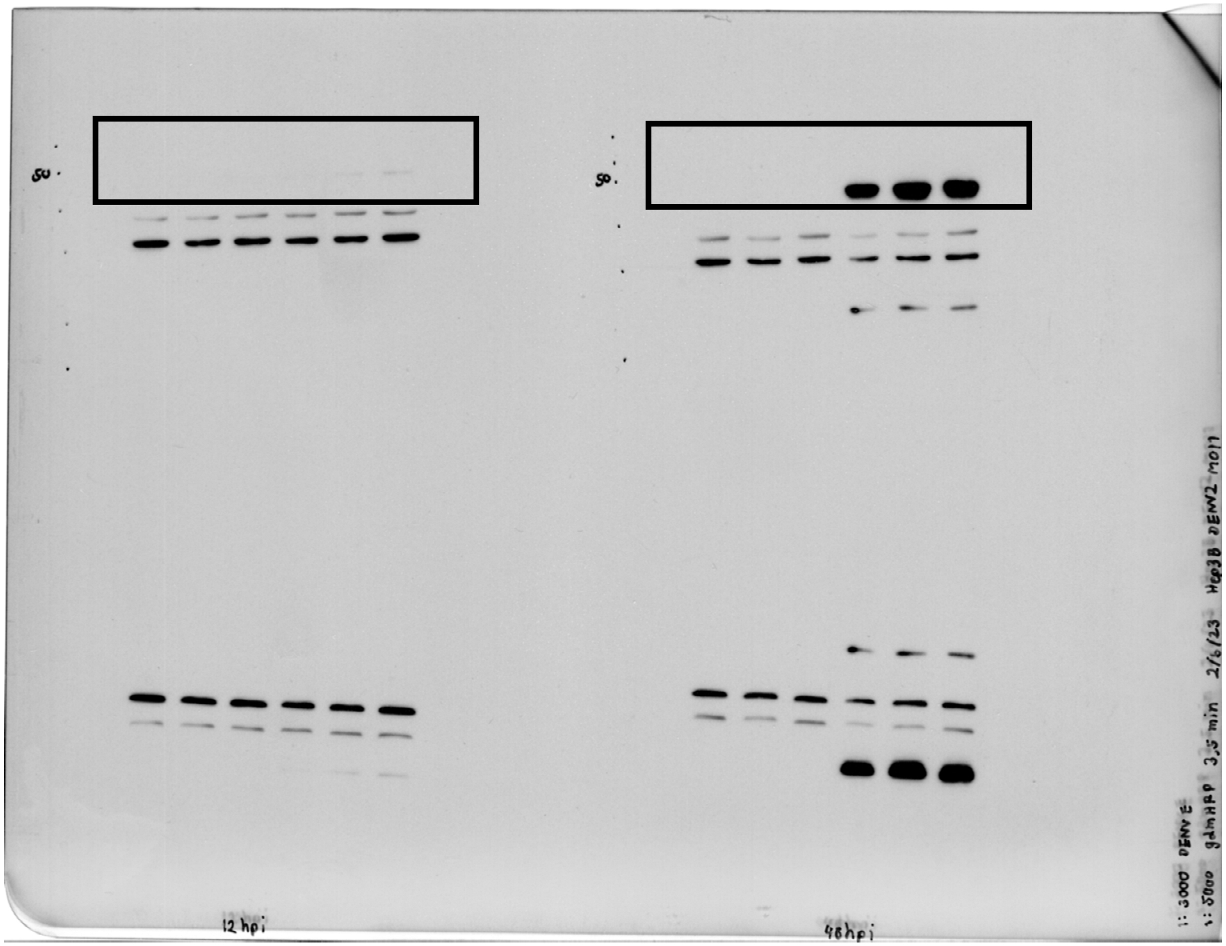
50
50
12 hpi
48hpi
1: 3000 DENV E
1: 5000 gdmHRP 3.5 min 2/6/23 Hep3B DENV2 MOI1

Fig 3B:
(actin)

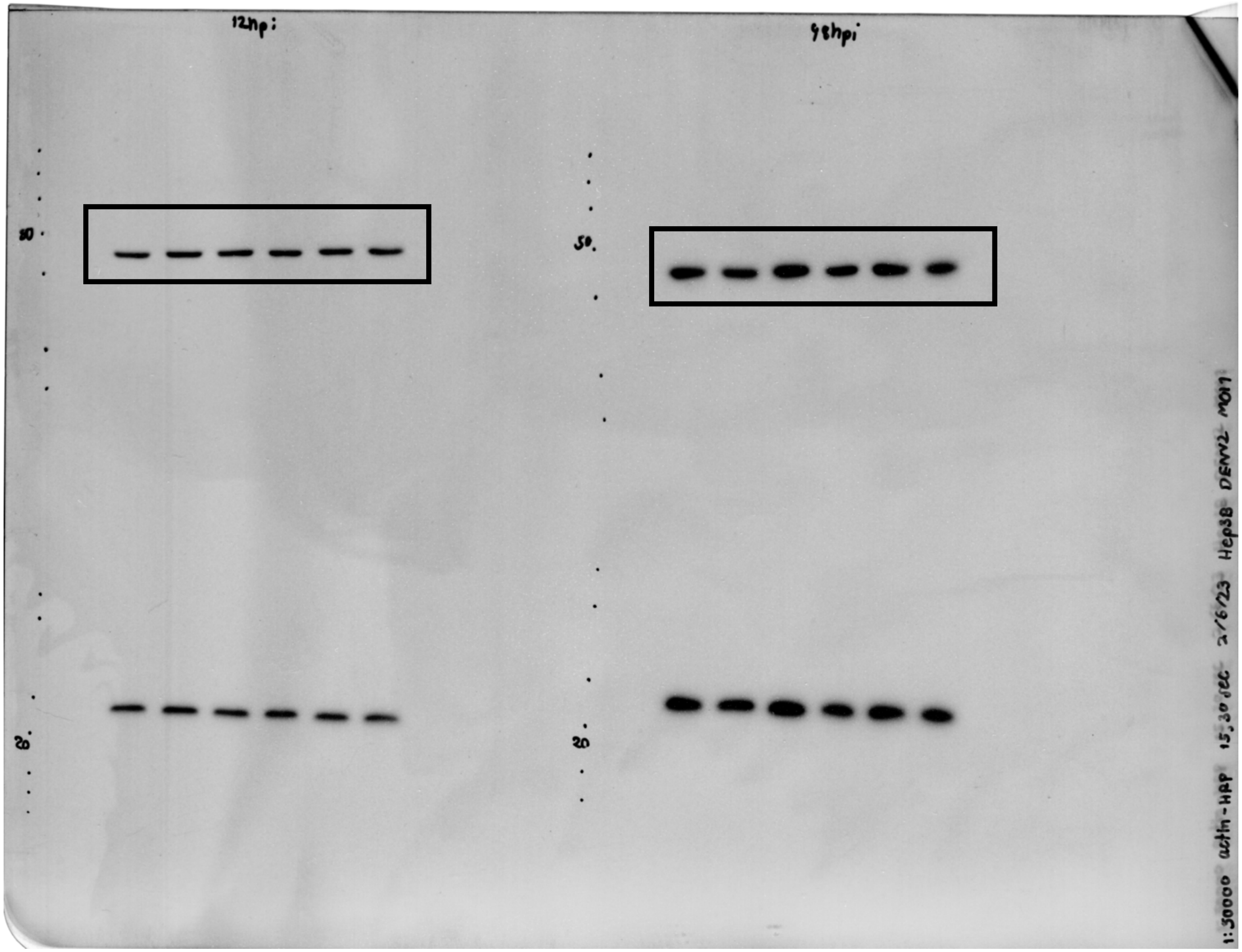
12hpi
48hpi
50
20
50
20
1:30000 actin-HRP 15,30 sec 2/6/23 Hep3B DENV2 MOI1

Fig 4D:
(GAPDH)

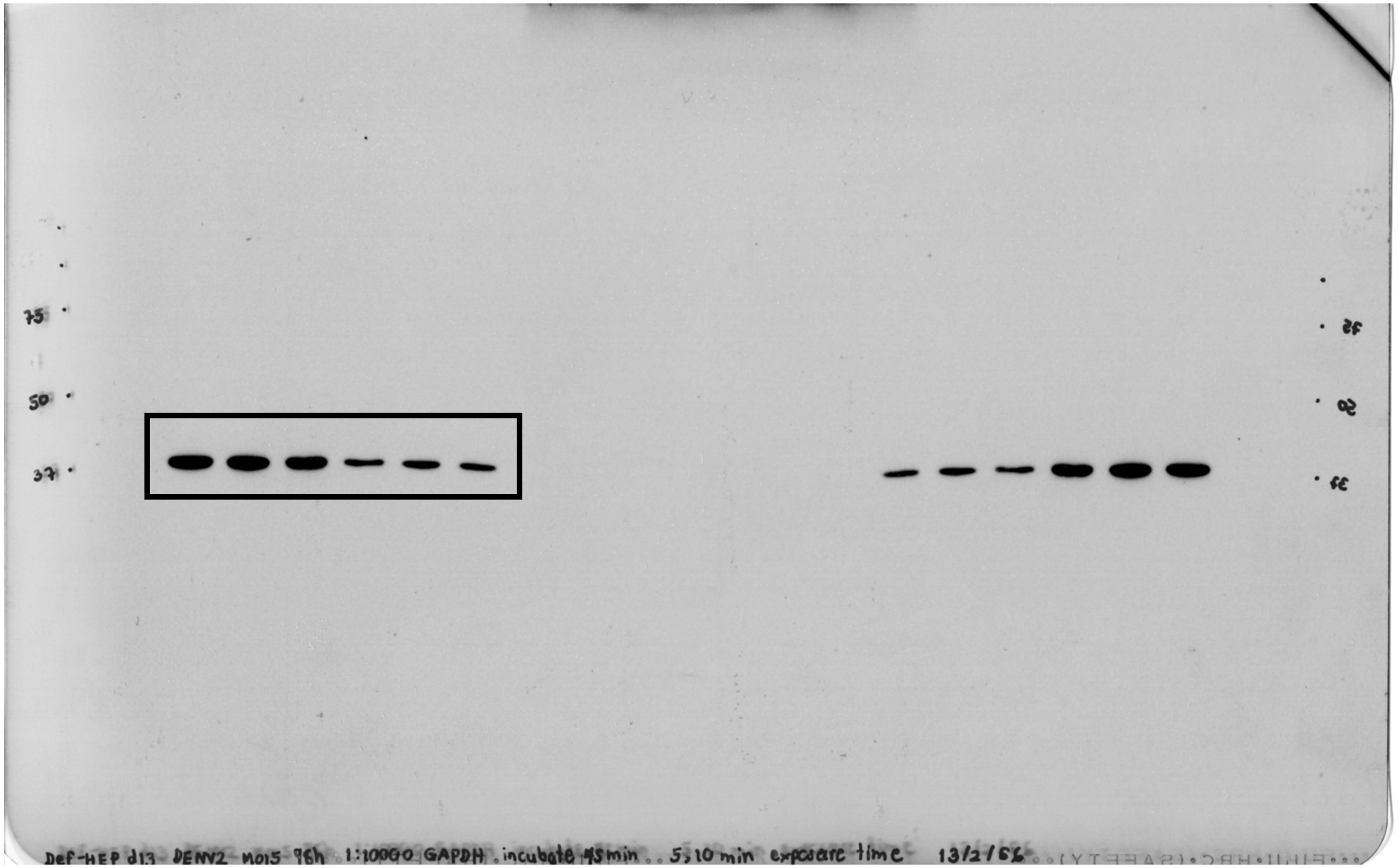
75
50
37
Def-HEP d13 DENV2 MOI5 96h 1:10000 GAPDH incubate 45min 5,10 min exposure time 13/2/66

Fig 4D:
(DENV E)

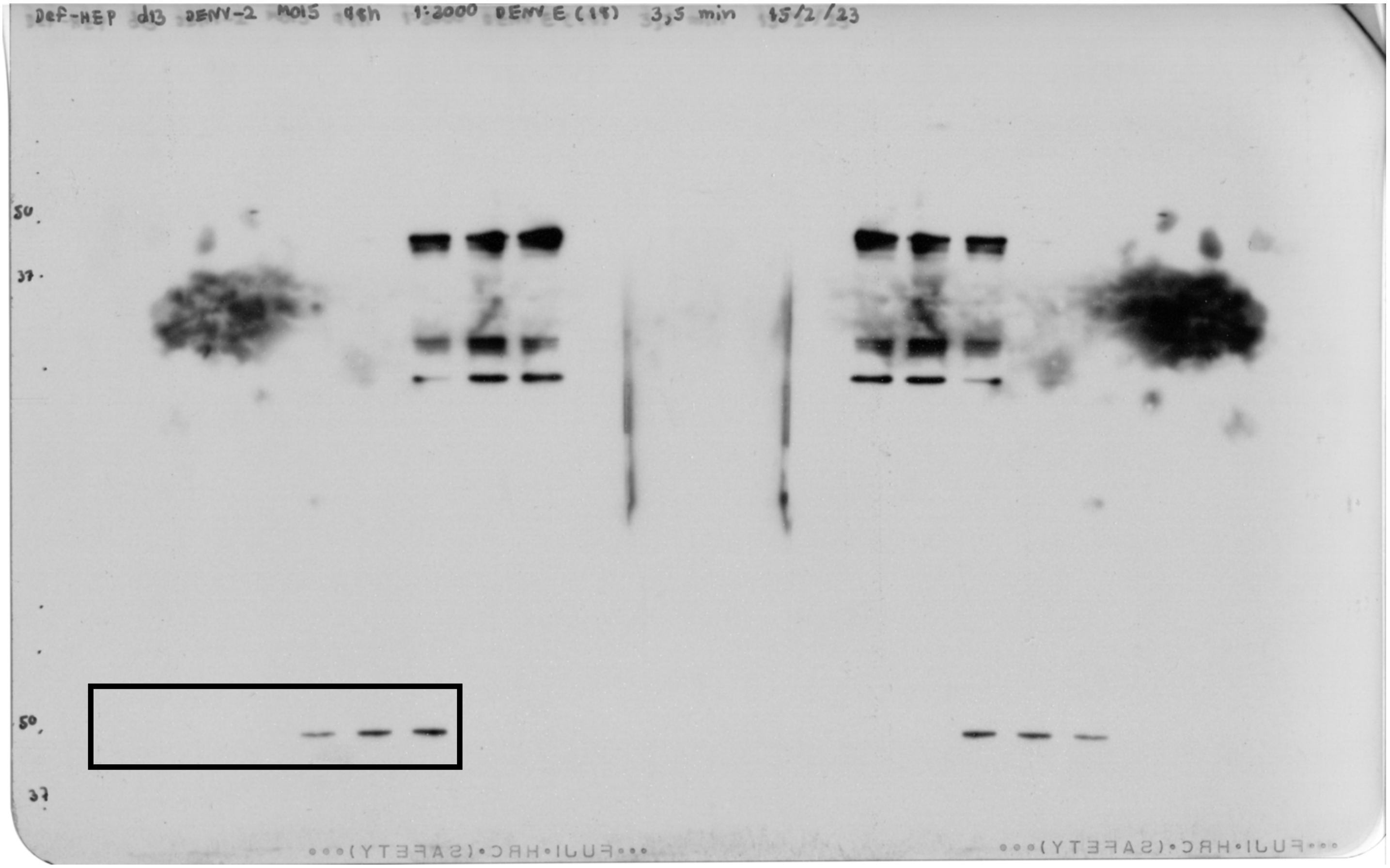

Fig 4D:
(DENV NS1)

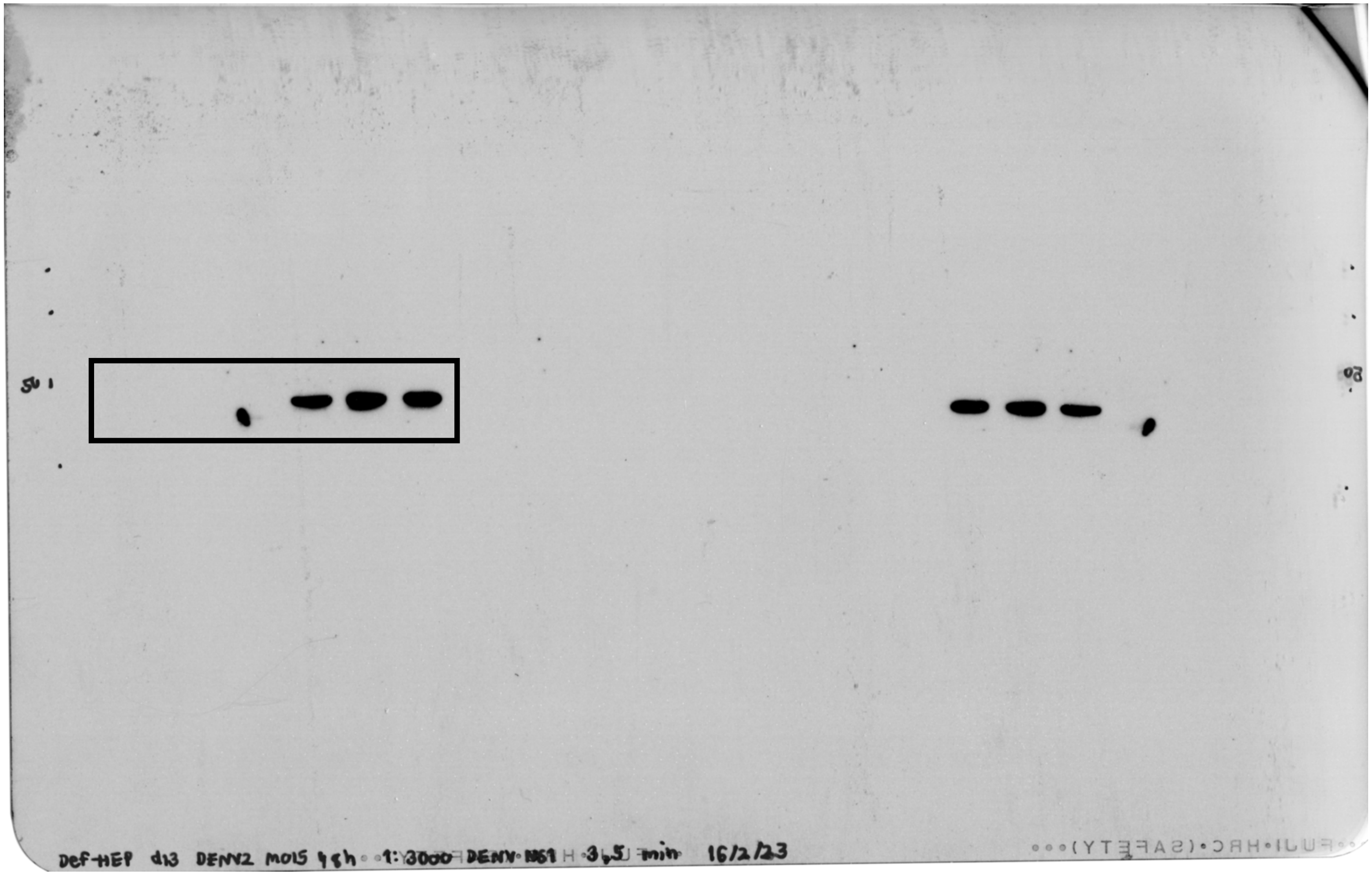

Fig 4D:
(actin)

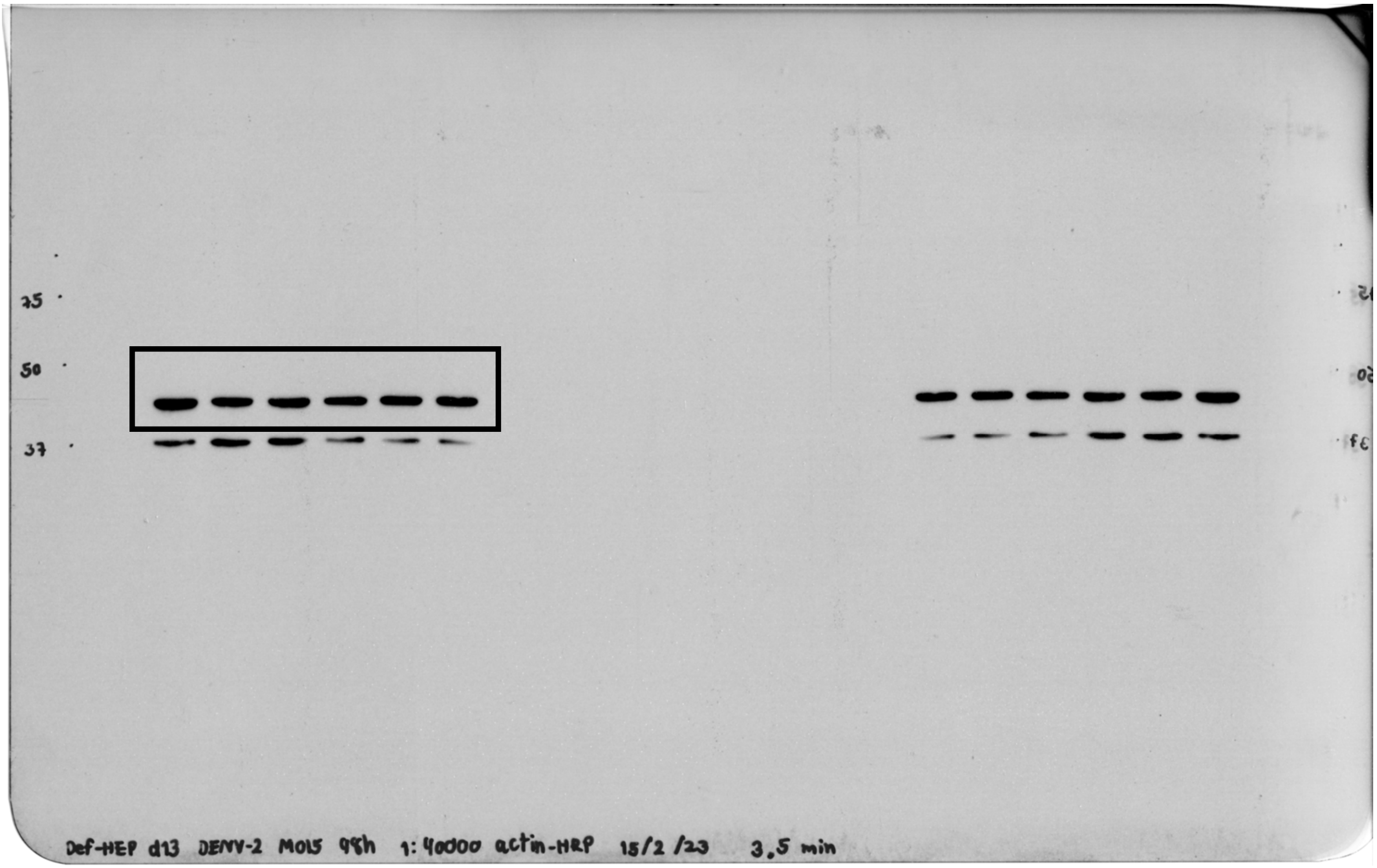

75
50
37
Def-HEP d13 DENV-2 MOI5 48h 1:40000 actin-HRP 15/2/23 3,5 min

Fig 5F:
(DENV E)

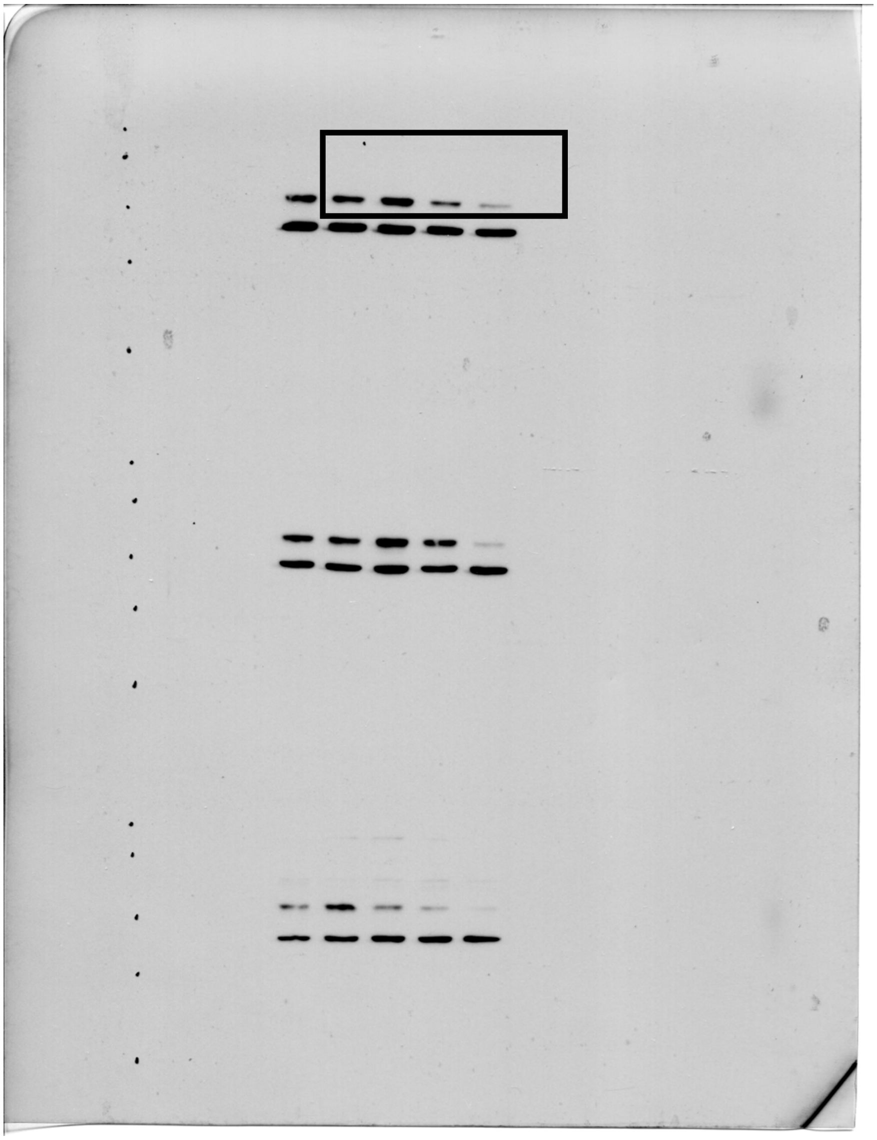

Fig 5F:
(DENV NS1)

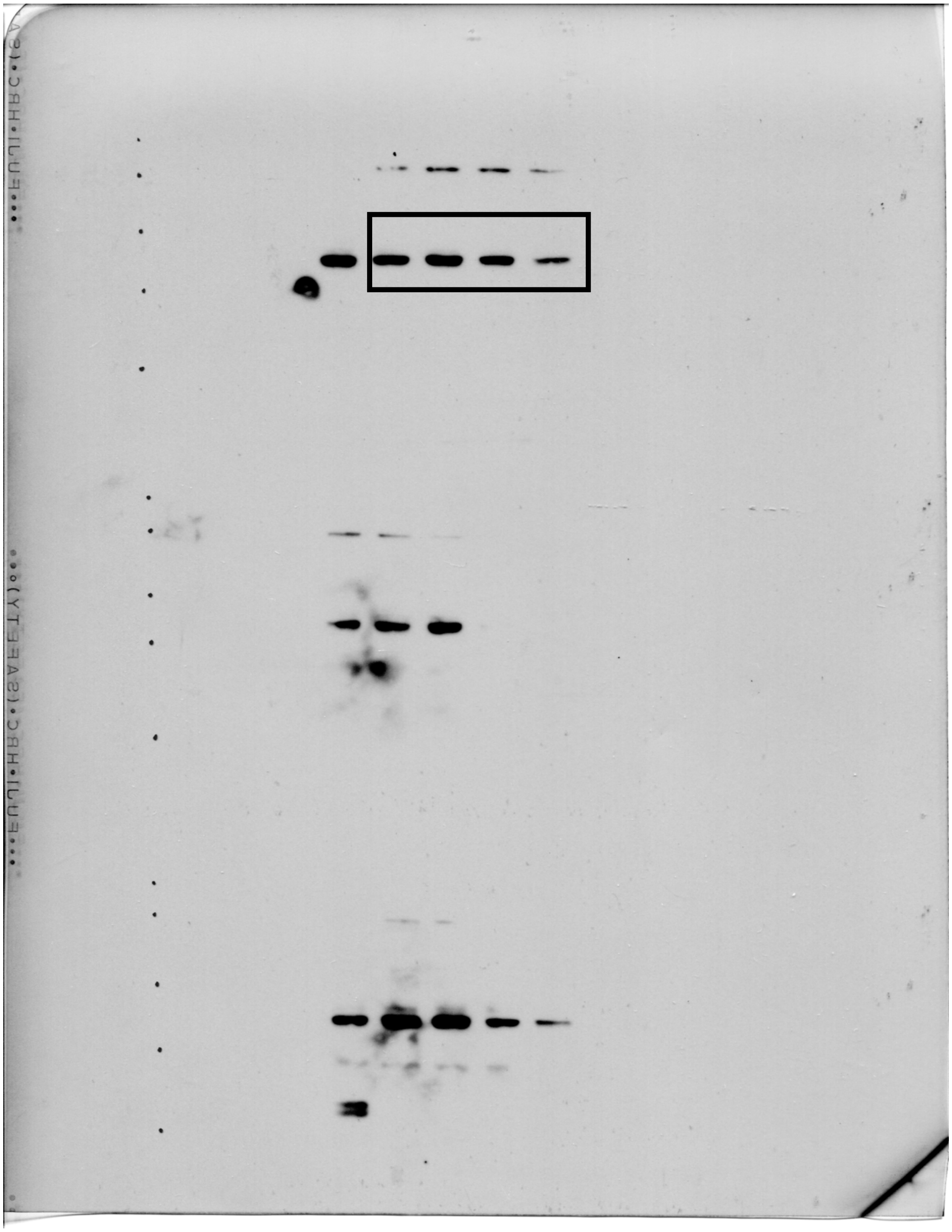

Fig 5F:
(DENV NS5)

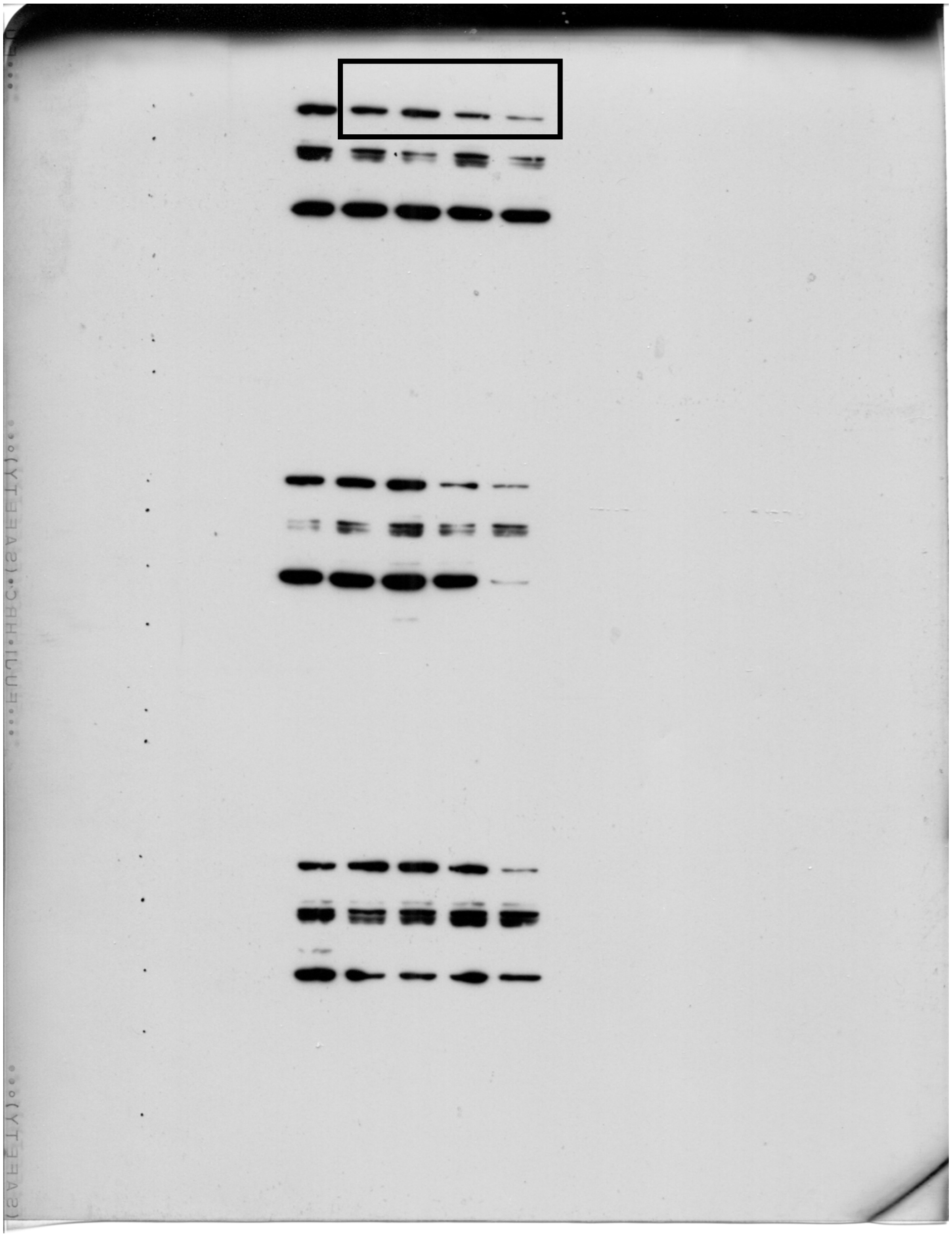

Fig 5F:
(actin)

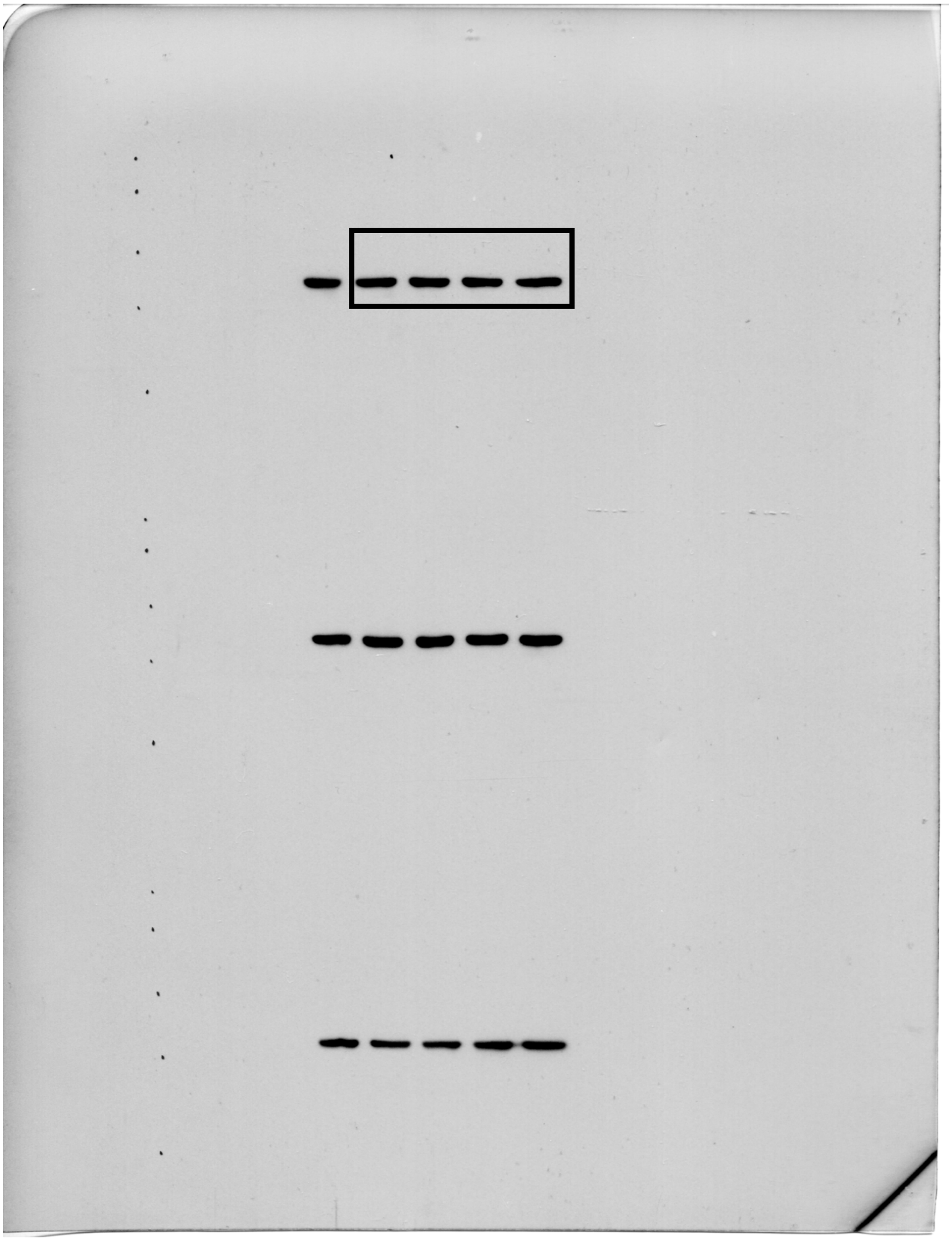

Fig : S8

(Mock GAPDH)

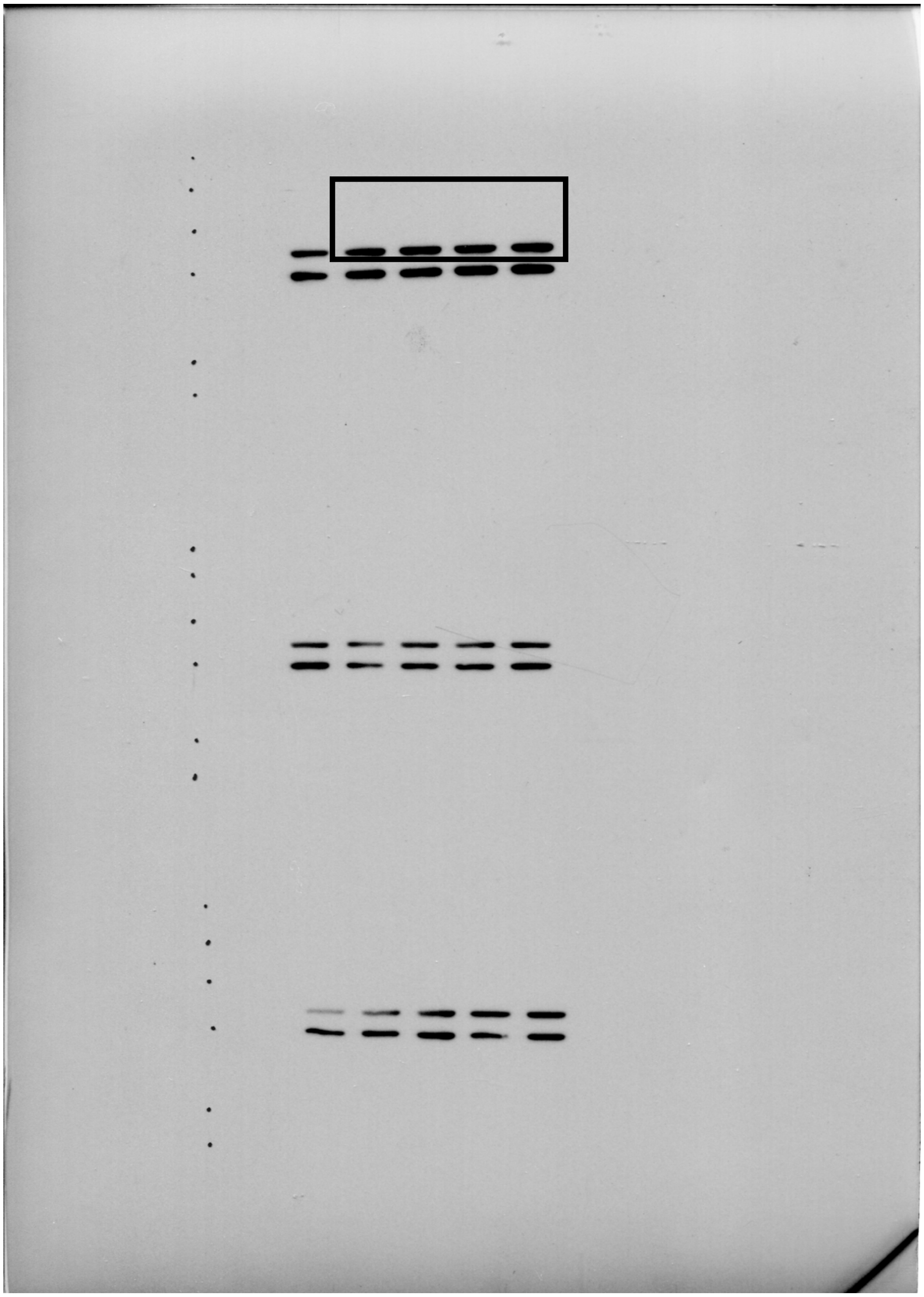

Fig : S8
(Mock Actin)

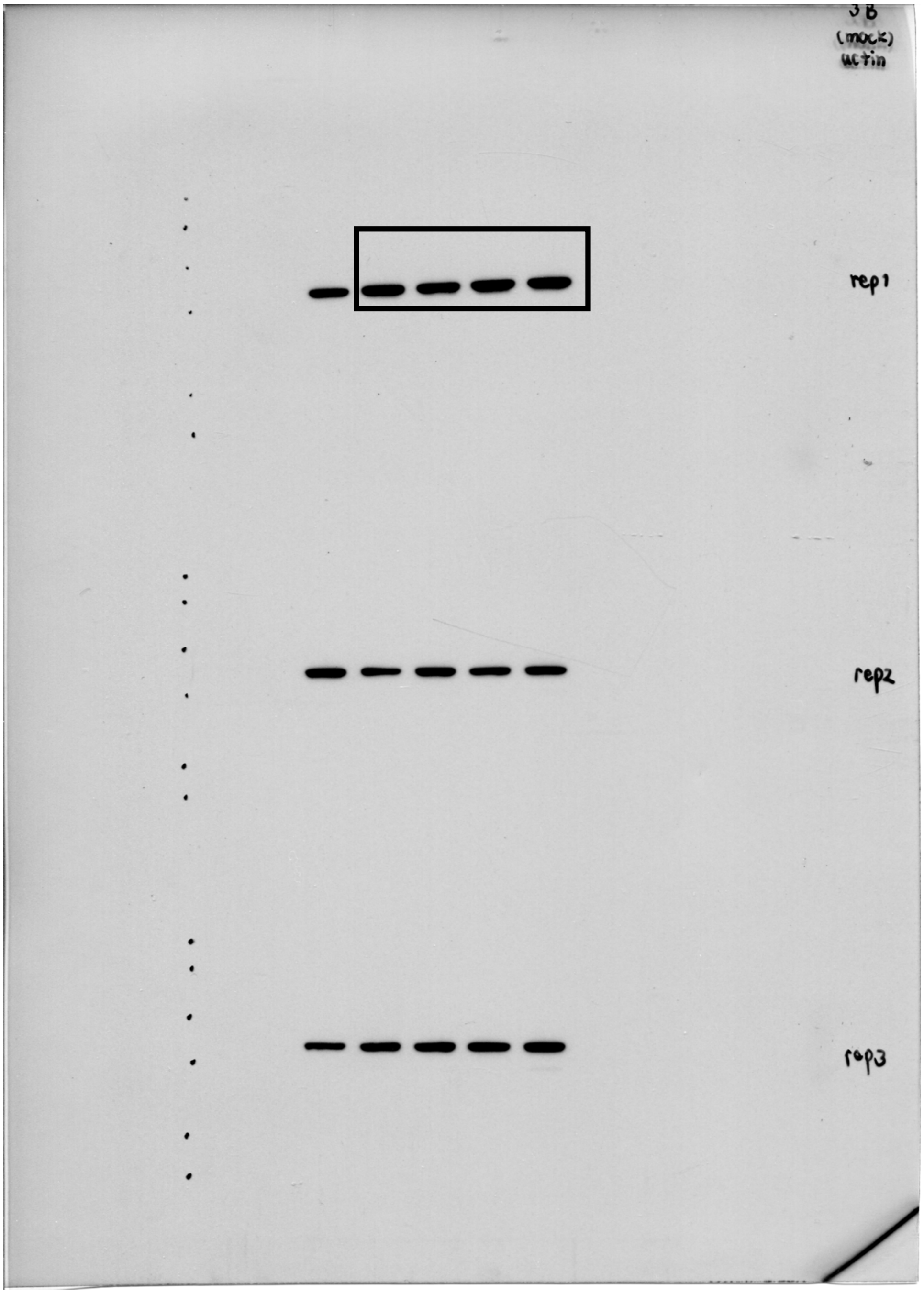

Fig :S8
(DENV 2 GAPDH)

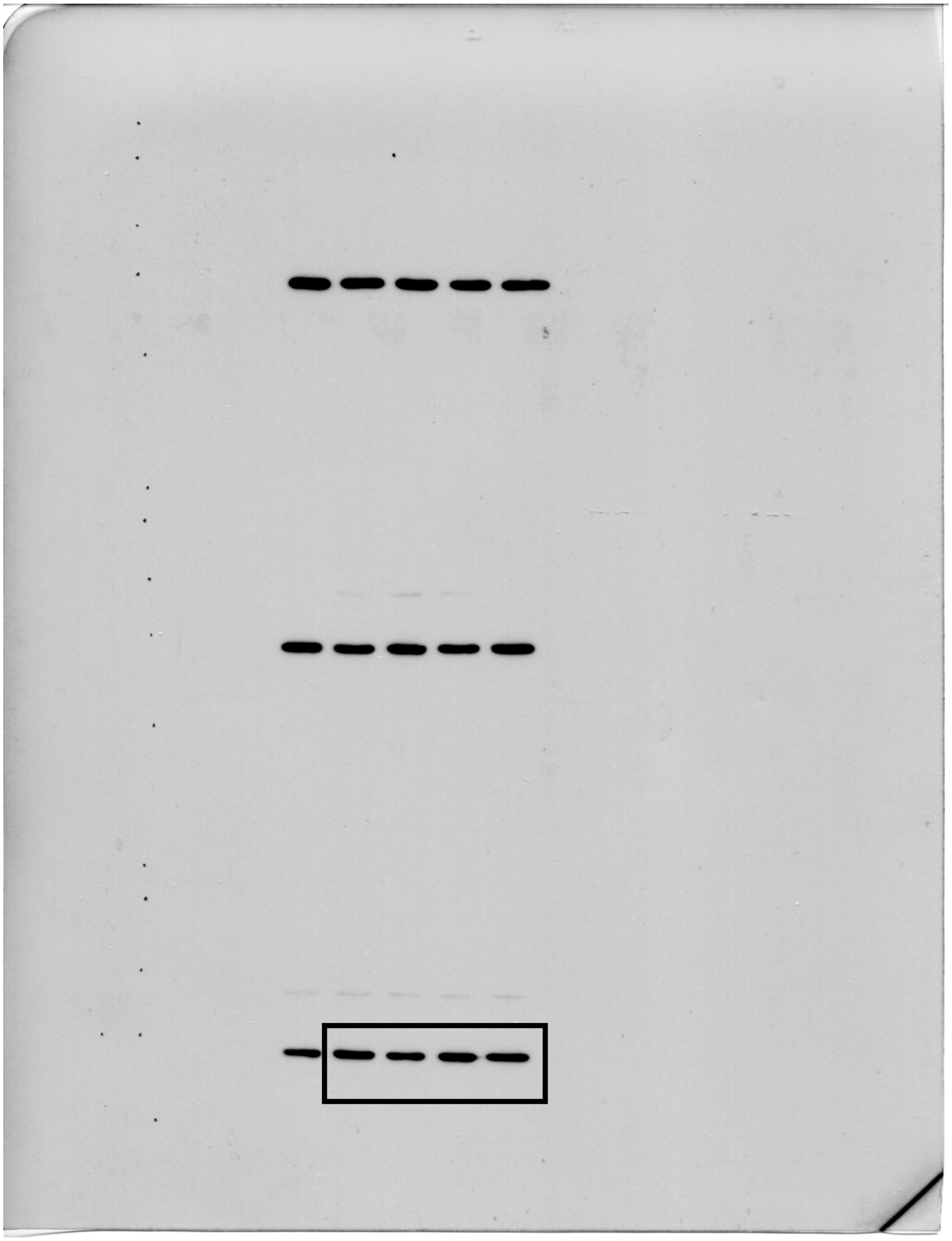

Fig : S8
(DENV 2 Actin)

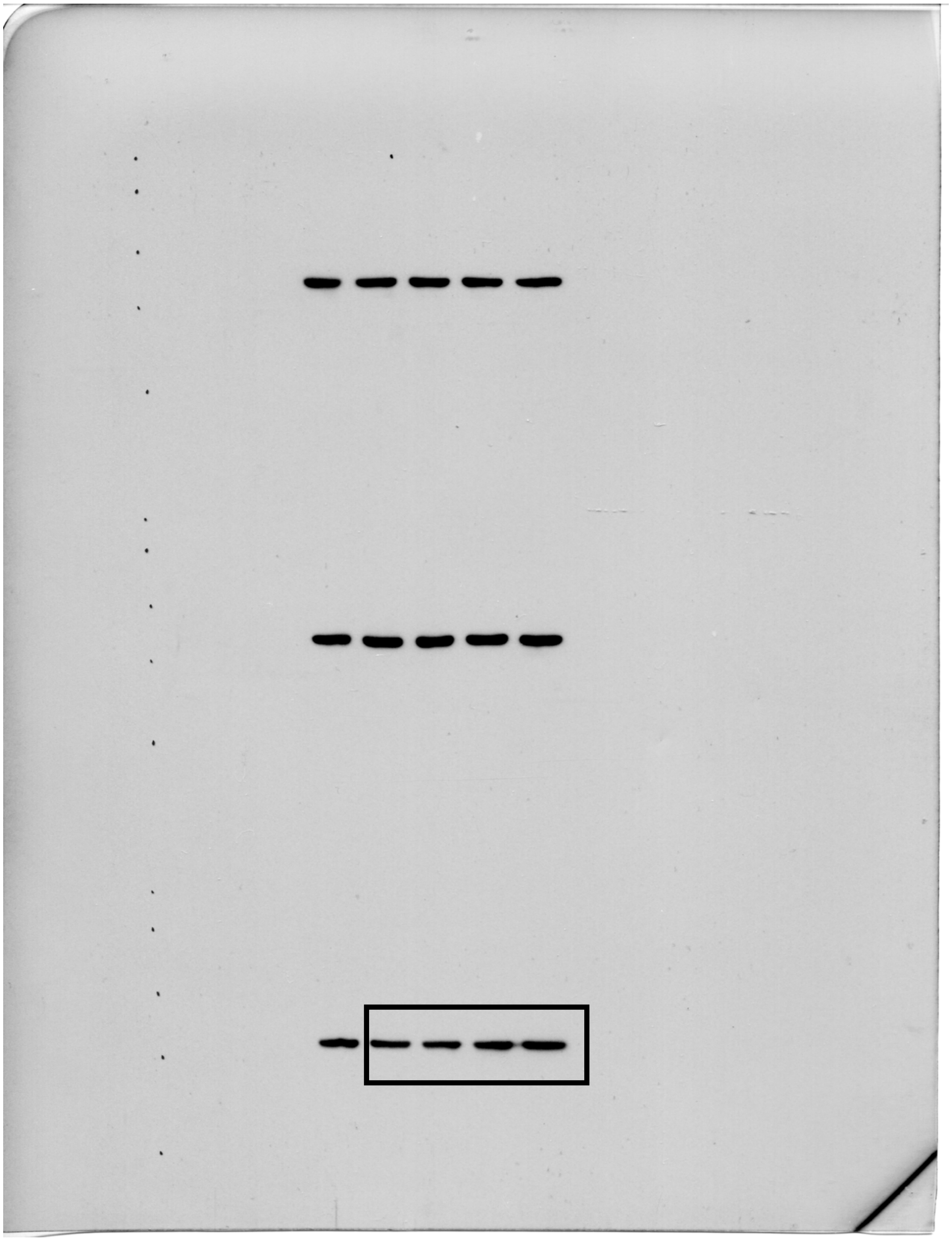

Supplement: Supplementary file 1 — Supplementary Information. [file 41598_2024_58834_MOESM1_ESM.pdf]
